# Intestinal lymphatic vasculature is functionally adapted to different drainage regions and is altered by helminth infection

**DOI:** 10.1084/jem.20241181

**Published:** 2025-06-12

**Authors:** Jorden I. Lane, Elida Nieves-Ortiz, Ornella Ndatabaye, Aliia R. Fatkhullina, Sebastian Lopez, Terence S. Dermody, Daria Esterházy

**Affiliations:** 1 Department of Pathology, University of Chicago, Chicago, IL, USA; 2 Department of Pediatrics, University of Pittsburgh School of Medicine, Pittsburgh, PA, USA; 3 Department of Microbiology and Molecular Genetics, University of Pittsburgh School of Medicine, Pittsburgh, PA, USA; 4 Institute of Infection, Inflammation, and Immunity, UPMC Children’s Hospital of Pittsburgh, Pittsburgh, PA, USA

## Abstract

We sought to determine whether the lymphatic vasculature functionally adapts to the organ in which it resides, such as along the gut. Duodenal lymphatic capillaries (lacteals) displayed the most discontinuous tight junction composition within the gut, resulting in a dependence on duodenal lacteals for rapid dietary lipid uptake. Duodenal helminths abrogated these features. Parallel RNA sequencing of lymphatic endothelial cells and mucosa along the intestine revealed that the transcriptomes overlapped in functional profiles. RNA sequencing also identified a putative VEGFR-2/3 signaling gradient that may explain differences in lacteal tight junctions along the small intestine at homeostasis. Transcriptionally, helminth infection triggered antimicrobial and angiogenic responses. While microbial depletion acted additively to helminths on lymphatic restructuring, glucocorticoids partially reversed helminth-induced lacteal changes. This suggests helminths induce lymphangiogenesis and associated lymphatic “zippering” via inflammation. Our study uncovers and explains the superior lipid absorption by duodenal lacteals and how it is compromised by helminths and provides transcriptional insights into lymphatic function along the gut.

## Teaser

Lane et al. discover that the lymphatic vasculature is functionally and transcriptionally distinct along the gut. Duodenal lymphatic vessels appear optimized for dietary lipid uptake. Duodenal helminths compromise this property, providing an alternative explanation for worm-induced resistance to weight gain.

## Graphical Abstract

**Figure:**
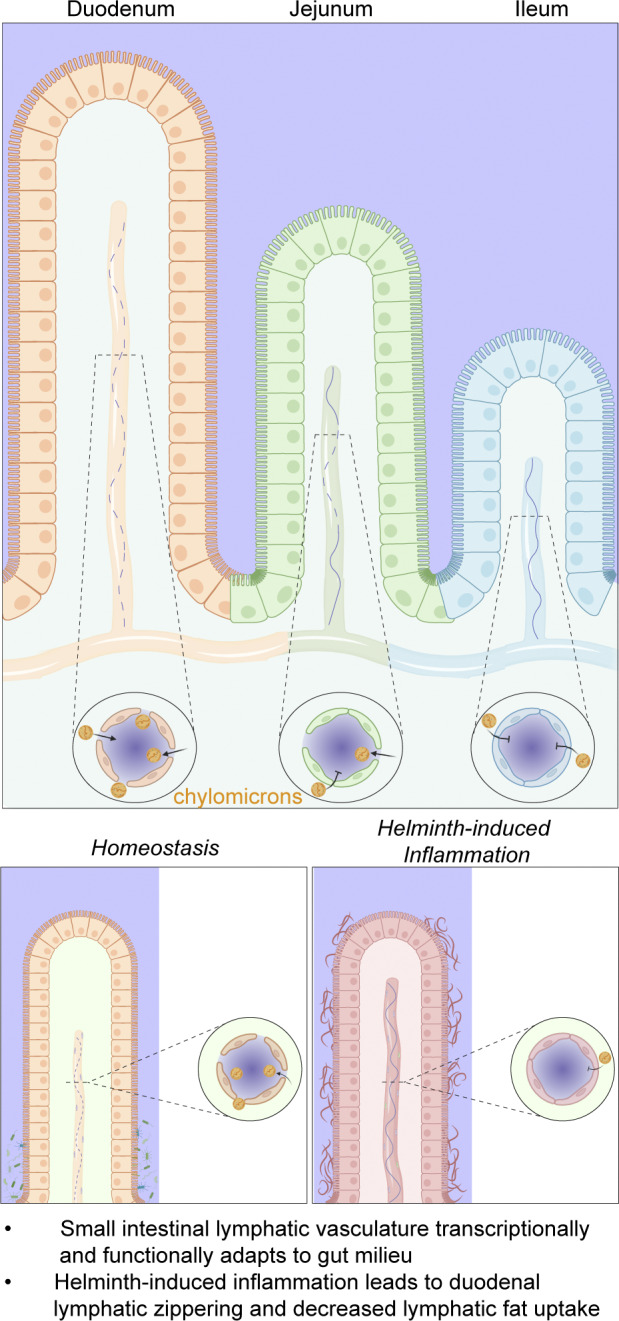

## Introduction

The lymphatic system universally serves the crucial functions of fluid recuperation and immune cell trafficking (Petrova and Koh, 2020). It fulfills these tasks by a generic principle, whereby initial lymphatic capillaries in organs drain interstitial fluid, feeding into larger lymphatic collectors. Valves and surrounding smooth muscle propel lymph unidirectionally to the draining lymph nodes (LNs), after which lymph collects in the thoracic duct and ultimately returns to the bloodstream (Oliver et al., 2020). Given the lymphatic vasculature acts as a conduit for immune cells to traffic to and from LNs, it facilitates the initiation of site-specific adaptive immunity in mammals and a few birds, the only organisms with LNs.

Lymphatic vasculature dysfunction has been associated with various diseases, most notably lymphedema, obesity, cancer, Crohn’s disease, and neurodegeneration (Oliver et al., 2020), and more recently has been recognized to play a role in infection (Yao et al., 2012; Loo et al., 2017). Despite the involvement of the lymphatic vasculature in these conditions, the cause and nature of the vascular defects are mostly ill-defined, which hampers the development of effective therapeutic approaches to treat them. Major limitations stem in part from a lack of in-depth understanding of tissue-specific homeostatic lymphatic endothelial functions and properties, although their existence is well accepted (Petrova and Koh, 2018). Evidence that the lymphatic vasculature adapts to the organ it drains comes from imaging studies of the lymphatic vasculature that describe a unique architecture and density within the skin (Karaman et al., 2015), kidney, pancreas (Cai et al., 2023), airways (Baluk et al., 2007), gut (Esterházy et al., 2019), and LNs (Xiang et al., 2020). In addition, transcriptional profiling of lymphatic endothelial cells (LECs) across select tissue sites has confirmed diverse immunological capacities such as chemokine secretion and antigen presentation (Tamburini et al., 2019; Berendam et al., 2019) and revealed highly nuanced heterogeneity within a single organ, with as many as six subclasses of LECs in LNs (Xiang et al., 2020; Abe et al., 2022).

Perhaps the most insightful organ in which to study the capacity of the lymphatic vasculature to adapt to a tissue is the gastrointestinal tract, a highly complex and fluctuating environment. Indeed, the capillaries in the small intestine, called lacteals, protrude into every single villus and serve the unique additional function of absorption of lipids and other hydrophobic molecules (Bernier-Latmani and Petrova, 2017). In neonatal mice, this capacity has been linked to exposure to lipids (Zarkada et al., 2023); in adult mice, a high-fat diet (HFD) regulates lacteal regeneration (Bernier-Latmani et al., 2015). LECs close to the villus crypts are essential for maintenance of the intestinal stem cell niche (Niec et al., 2022). The colon, the major site of fluid reabsorption from the lumen, has a unique two-layered lymphatic system that differs from that of the small intestine (Esterházy et al., 2019). The absence of a microbiota leads to an elongation of duodenal lacteals and blunting of those in the ileum (Esterházy et al., 2019), the latter of which is also seen following acute microbial depletion (Suh et al., 2019). Conversely, infection with *Yersinia pseudotuberculosis* leads to a breach of the mesenteric collecting vessels, causing chylous ascites and inflammation of the mesenteric fat (Fonseca et al., 2015), a phenotype resembling the peri-intestinal “creeping fat” in Crohn’s disease (Mao et al., 2019; Alexander et al., 2010). LECs in the LNs along the gut display differential expression patterns that align with the immunological function of each LN (Esterházy et al., 2019). However, while all these features strongly point to an influence of the local environment on lymphatics, it is not known whether the lymphatic system functionally differs along the intestine, such as in its dietary lipid uptake capacity and immunological properties, what the molecular underpinnings are, how the vasculature responds to local perturbations, and how these factors may ultimately shape immune events in the draining LN.

In the current study, we sought to start addressing some of these knowledge gaps. Specifically, we asked whether the absorptive capacity of the lymphatic vasculature along the small intestine varied, if so, whether this was altered by local infections, and more broadly, how any functional absorptive and structural differences we found could be explained by transcriptional profiles in the LECs and surrounding mucosa.

## Results

### Tissue lymphatic vasculature is structurally distinct along the small intestine and differs in absorptive capacity

**Figure 1.**
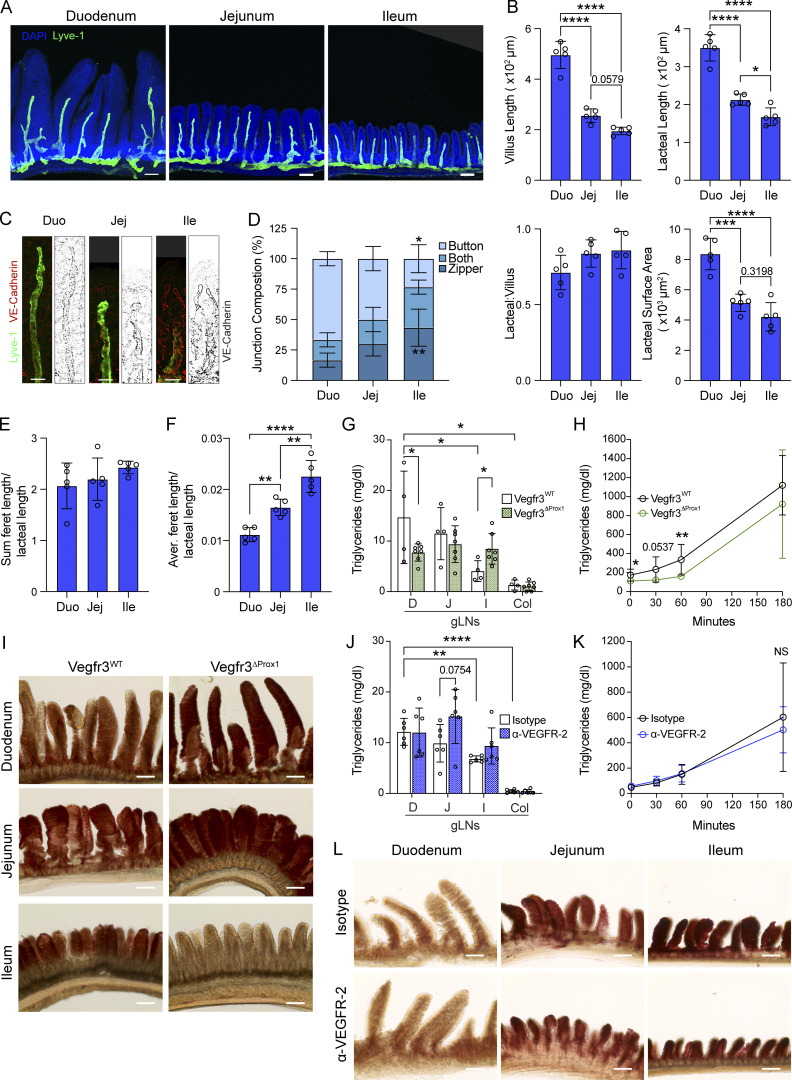
**Tissue lymphatic vasculature is structurally and functionally different along the gut. (A and B)** Immunofluorescence images (A) and comparisons of villi and LYVE-1+ lacteals along the small intestine (SI) (B): villus length, lacteal length, lacteal-to-villus length ratio, and lacteal surface area along the small intestine in WT C57BL/6 mice (*n* = 5). Scale bar: 100 μm. Data are representative of two independent experiments. **(C–F)** Representative images (C) and characterization of VE-cadherin+ LEC junctions of LYVE-1+ lacteals along the SI in C57BL/6 mice. Quantification of SI lacteal tight junction organization with primarily button-like, zipper-like, or a combination of both tight junction formations throughout the length of the lacteal (D), sum of VE-cadherin+ junction (feret) lengths of LYVE-1+ lacteals normalized to the length of the lacteal (E), and mean VE-cadherin+ feret length of LYVE-1+ lacteals normalized to the length of the lacteal (F) (*n* = 5). Scale bar: 25 μm. Data are representative of two independent experiments. **(G–I)** Intestinal-draining LN (G), plasma triglyceride concentration over time (H), and representative images of Oil Red O staining (I) of intestinal villi 3 h after olive oil challenge in the presence of poloxamer of Vegfr3^ΔProx1^ and Vegfr3^WT^ littermates (*n* = 4–7). Data are pooled from two independent experiments. Scale bar: 125 μm. **(J–L)** Intestinal LNs (J), plasma triglyceride concentration over time (K), and representative images of Oil Red O staining (L) of intestinal villi 3 h after olive oil challenge in the presence of poloxamer of mice treated with anti-VEGFR-2 or IgG2a isotype control antibody (*n* = 6). Data are pooled from two independent experiments. Scale bar: 125 μm. Abbreviations: duodenum (D), jejunum (J), ileum (I), and Cecal-colonic (Col). Error bars indicate the mean ± SD and *P < 0.05, **P < 0.01, ***P < 0.001, ****P < 0.001 by one-way ANOVA with Šídák’s multiple comparisons test (B, D–G, and J) or two-tailed Student’s *t* test (comparison between genotypes [G and H] or treatments [J and K] at single locations or time points). Data are represented as the mean ± SD. Duo, duodenum; Jej, jejunum; Ile, ileum.

**Figure S1.**
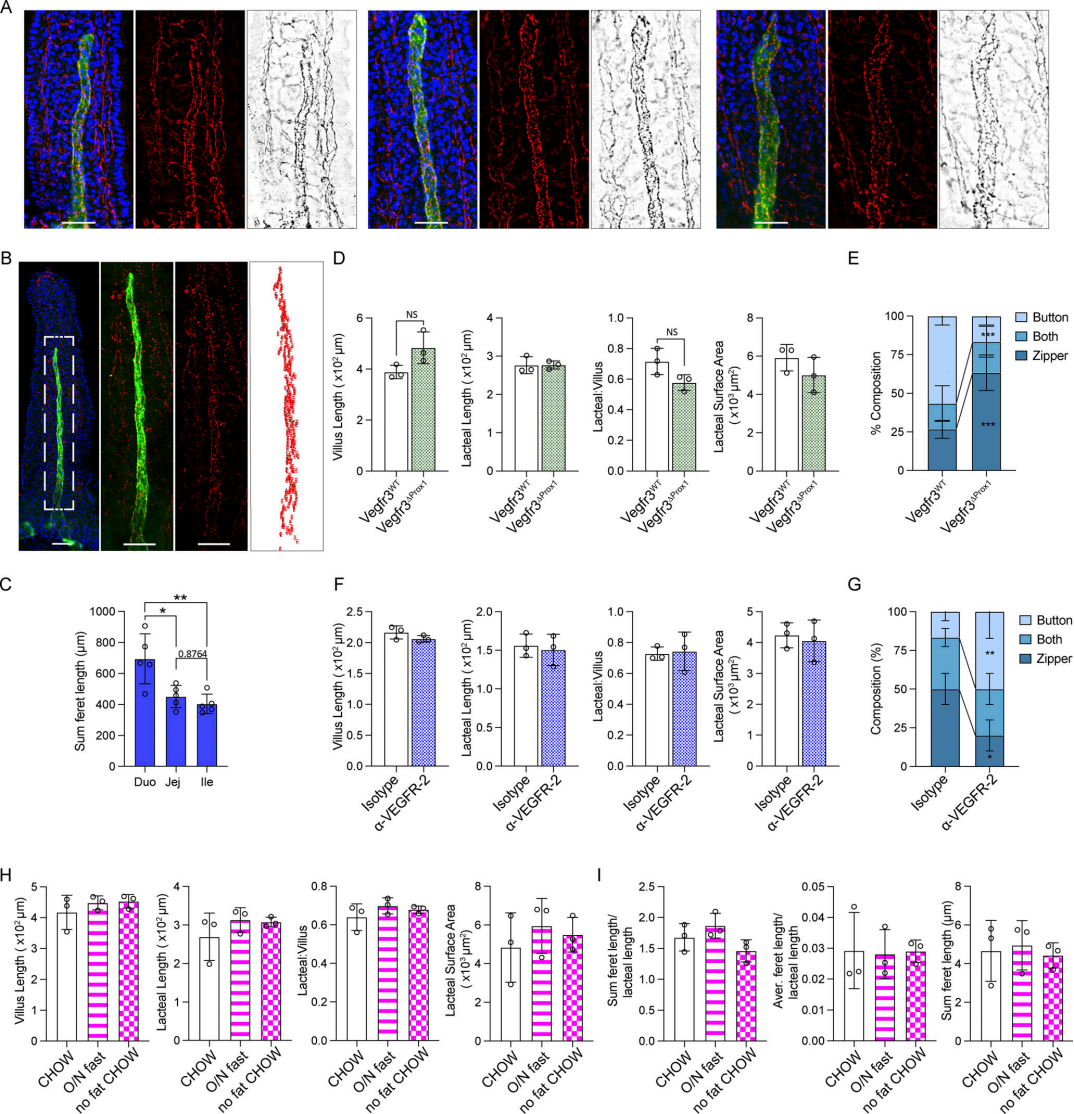
**Duodenal lacteal morphology following VEGF receptor signaling blockade and dietary intervention** **. (A and B)** Immunofluorescence images of villi stained for LYVE-1 (green) and VE-cadherin (red or gray) and counterstained with DAPI and representative of lacteal classified as displaying zipper-like tight junctions (left), a mix of zippers and buttons (“both,” center), and mostly button-like junctions (right) (A) or representative of a lacteal of which VE-cadherin staining within LYVE-1+ area was subjected to automated feret assignment (B). Scale bars = 100 μm. **(C)** Sum feret length per lacteal along small intestine (*n* = 5). Data are representative of one experiment. **(D)** Duodenal villus length, lacteal length, lacteal-to-villus length ratio, and lacteal surface area of Vegfr3^ΔProx1^ and Vegfr3^WT^ mice (*n* = 3). Data are representative of one experiment. **(E)** Quantification of duodenal lacteal tight junction organization with primarily button-like, zipper-like, or a combination of both tight junction formations throughout the length of the duodenal lacteal of Vegfr3^ΔProx1^ and Vegfr3^WT^ mice (*n* = 3). Data are representative of one experiment. **(F)** Ileal villus length, lacteal length, lacteal-to-villus length ratio, and lacteal surface area of mice treated with anti-VEGFR-2 or IgG2a isotype control antibody (*n* = 3). Data are representative of one experiment. **(G)** Quantification of duodenal lacteal tight junction organization with primarily button-like, zipper-like, or a combination of both tight junction formations throughout the length of the duodenal lacteal of mice treated with anti-VEGFR-2 or IgG2a isotype control antibody (*n* = 3). Data are representative of one experiment. **(H and I)** Duodenal villus length, lacteal length, lacteal-to-villus length ratio, and lacteal surface area (H) and sum of VE-cadherin+ junction (feret) lengths of LYVE-1+ lacteals normalized to the length of the lacteal, mean VE-cadherin+ feret length of LYVE-1+ lacteals normalized to the length of the lacteal, and total feret length (I) in mice fed a chow diet, fasted overnight (O/N), or fed a diet without added fats (*n* = 3); data are representative of one experiment. Error bars indicate the mean ± SD and *P < 0.05, **P < 0.01 by two-tailed Student’s *t* test (D–G) or two-way ANOVA with Šídák’s multiple comparisons test (C, H, and I).

We first determined whether the lymphatic absorptive properties change along the small intestine. The architecture of the lymphatic system along the intestine has been examined previously, and notably, the lacteals, like the villi, shorten along the small intestine. The lacteals can appear branched in the duodenum and jejunum, yet reach further into the villus tip in the ileum (Bernier-Latmani et al., 2015, Fig. 1, A and B). Unsurprisingly, the lacteal (LYVE-1+) surface area is also the largest in duodenal and smallest in the ileal villi (Hong et al., 2020, Fig. 1 B). Since lymphatic absorptive capacity is primarily dictated by permeability, we assessed LEC tight junction organization using tight junction VE-cadherin staining (Fig. 1 C). As established by others (Baluk et al., 2007; Zhang et al., 2018; Bernier-Latmani et al., 2015), we categorized lacteals as containing primarily impermeable “zipper-like” junctions, permeable “button-like” junctions, or a mix of both (Fig. S1 A). We found that lacteals were mostly button-like in the duodenum and became increasingly more zipper-like toward the ileum (Fig. 1 D). We sought to confirm this with an independent, automated method measuring the length of VE-cadherin+ junctions (ferets) within the LYVE-1+ area. This analysis revealed that the average tight junction length increased from proximal to distal small intestine (Fig. 1, E and F; and Fig. S1, B and C). Together, these results suggest a lacteal permeability gradient along the small intestine.

To determine whether this tight junction gradient impacts lipid uptake, we gavaged mice with a bolus of olive oil and measured plasma and gut-draining LN (gLN) triglyceride content over and after 3 h, respectively. High-dose olive oil reaches all gut segments in the course of 3 h, but since chylomicron formation efficiency along the small intestine may also differ, we coupled this analysis to a comparison of mice in which we induced the loss of *Vegfr3* expression in LECs of adult mice using the Prox1Cre^ERT2^ driver (Vegfr3^ΔProx1^) (Jannaway et al., 2023), which we confirmed to lead to a more zipper-like LEC tight junction organization in the duodenum (Jannaway et al., 2023) (Fig. S1, D and E). Conversely, we also coupled this study to mice treated with a VEGFR-2 blocking antibody (Churchill et al., 2022), which as predicted (Churchill et al., 2022) led to a more button-like LEC tight junction organization in the ileum (Fig. S1, F and G). Lipids accumulated in the gLNs in a decreasing proximal-to-distal gradient in wild-type (Vegfr3^WT^) littermate and isotype control antibody–treated mice (Fig. 1, G and J). Upon tamoxifen treatment, this gradient diminished in Vegfr3^ΔProx1^ mice, whereby the duodenal LNs accumulated fewer and the ileal LN more lipids (Fig. 1 G). Correspondingly, there was also a decrease in the plasma triglyceride content at 1 h after gavage in Vegfr3^ΔProx1^ compared with Vegfr3^WT^ littermate control mice (Fig. 1 H), a time point at which olive oil would only have reached the upper small intestine. Oil Red O staining of the gut also confirmed lipid stalling in the duodenum but more effective ileal clearance in the Vegfr3^ΔProx1^ mice (Fig. 1 I). This epithelial lipid accumulation resembles what has been observed when deletion of the HIPPO pathway in lamina propria stromal cells led to a zippering of lacteals and lipid stalling at the mucosa (Hong et al., 2020). In contrast, altering tight junctions to a more button-like permeable conformation using VEGFR-2 blocking antibody led to a slight increase in jejunal LN lipids (Fig. 1 J); despite this, plasma triglyceride content, lipid uptake kinetics (Fig. 1 K), and villus lipid accumulation (Fig. 1 L) were not impacted, likely because the primary site of lipid absorption, the duodenum, was not disturbed by this intervention. Finally, we interrogated if in adult mice lipid substrate availability is responsible for the more button-like conformation in duodenal lacteals by either placing mice on a chow diet with no added fats (0.5% calories from fat remaining from grain) or subjecting them to an overnight fast (~16 h) and comparing the lacteal morphology with fed mice placed on a regular chow diet (16% calories from fat). Of note, both interventions would result in luminal lipid levels lower than the ileum receives under fed chow conditions. Neither the low-fat diet nor the overnight fast changed duodenal lacteal morphology or tight junction length (Fig. S1, H and I), suggesting that maintenance of baseline duodenal lacteal architecture in adult mice does not require acute or chronic lipid exposure.

These results uncover the existence of a stable permeability and lipid uptake capacity gradient by the lacteals along the small intestine that mirrors the absorptive function of the gut segment they drain.

### Helminth infections alter duodenal lacteal morphology and absorptive function

**Figure 2.**
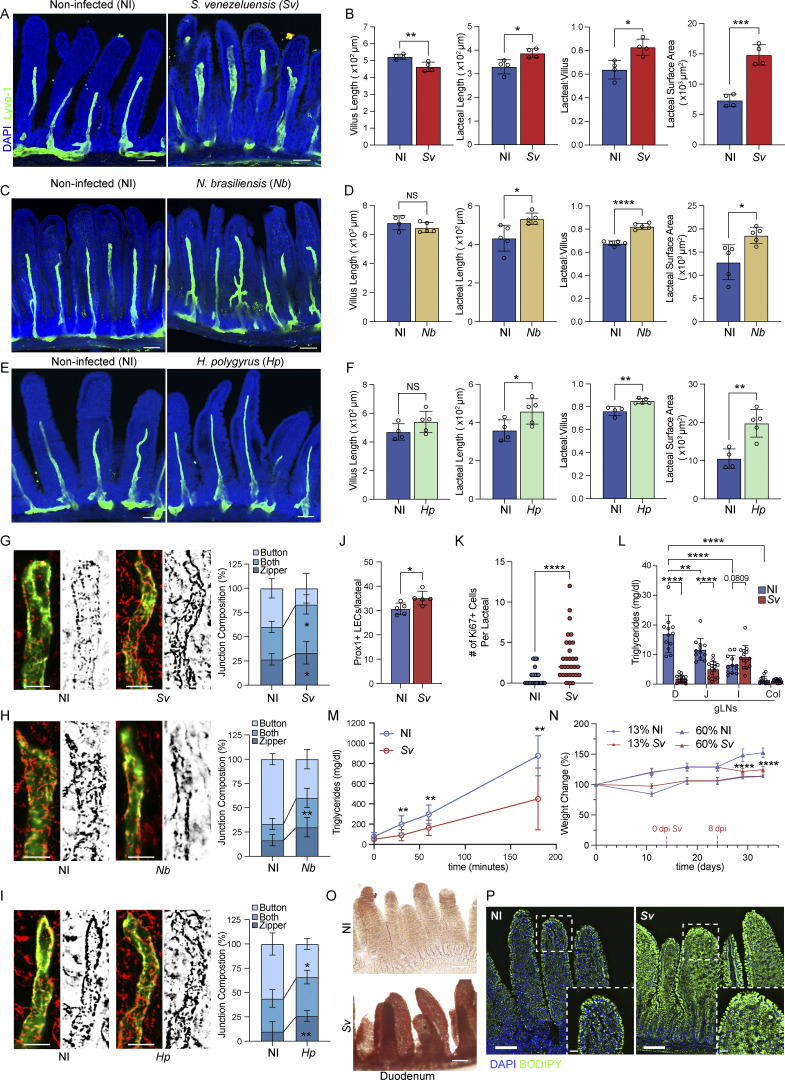
**Helminth infections impact duodenum lacteal morphology and function. (A–F)** Immunofluorescence images (A, C, and E) and comparisons of duodenum villi and LYVE-1+ lacteals in NI versus helminth-infected mice: villus length, lacteal length, lacteal-to-villus length ratio, and lacteal surface area 8 dpi with *Sv* (B), 8 dpi with *Nb* (D), and 14 dpi with *Hp* (F) (*n* = 3–4). Data are representative of one to two independent experiments. **(G–I)** Representative images and characterization of VE-cadherin+ LEC junctions of LYVE-1+ lacteals in NI versus helminth-infected mice. Quantification of SI lacteal tight junction organization with primarily button-like, zipper-like, or a combination of both tight junction formations throughout the length of the duodenal lacteal in NI mice versus mice infected with *Sv* 8 days (G), Nb 8 days (H), and *Hp* 14 days (I) earlier (*n* = 3–4). Data are representative of two independent experiments (B); data are representative of one experiment (D and F). **(J)** Quantification of DAPI+ Prox1+ nuclei within LYVE-1+ duodenum lacteals in NI versus mice infected with *Sv* 8 dpi (*n* = 5). Data are representative of one experiment. **(K)** Quantification of DAPI+ Ki67+ nuclei within LYVE-1+ duodenum lacteals in NI versus mice infected with *Sv* 8 dpi (*n* = 3). Data are representative of one experiment; each dot represents the number of Ki67+ nuclei per lacteal. **(L and M)** gLN (L) and plasma triglyceride concentration (M) over time after olive oil challenge in the presence of poloxamer of NI mice versus mice infected with *Sv* 8 dpi (*n* = 5 mice per group). Data are pooled from two independent experiments. **(N)** Growth curves of NI mice versus mice infected with *Sv* 2 wk after starting a 60% HFD or a 13% normal chow diet (*n* = 5). Data are representative of one experiment. **(O)** Representative images of Oil Red O staining of duodenal villi 3 h after olive oil challenge of NI mice versus mice infected with *Sv* 8 dpi. **(P)** Representative images of BODIPY-stained duodenal villi from NI and mice infected with *Sv* 8 dpi after ad libitum feeding. D, duodenum; J, jejunum; I, ileum; Col, colon; *Sv*, *S. venezuelensis*; *Nb*, *N. brasiliensis*; *Hp,H. polygyrus*; NI, noninfected; dpi, days post infection. Error bars indicate the mean ± SD and *P < 0.05, **P < 0.01, ***P < 0.001, ****P < 0.001 by two-tailed Student’s *t* test (comparison between conditions [B, D, F, J–L, and N] at single locations or time points) or two-way ANOVA (multiple comparisons between conditions within groups) (G–I). Scale bar: 100 μm (A, C, E, O and P), 10 μm (P inset), 25 μm (G–I), 125 μm (M).

**Figure S2.**
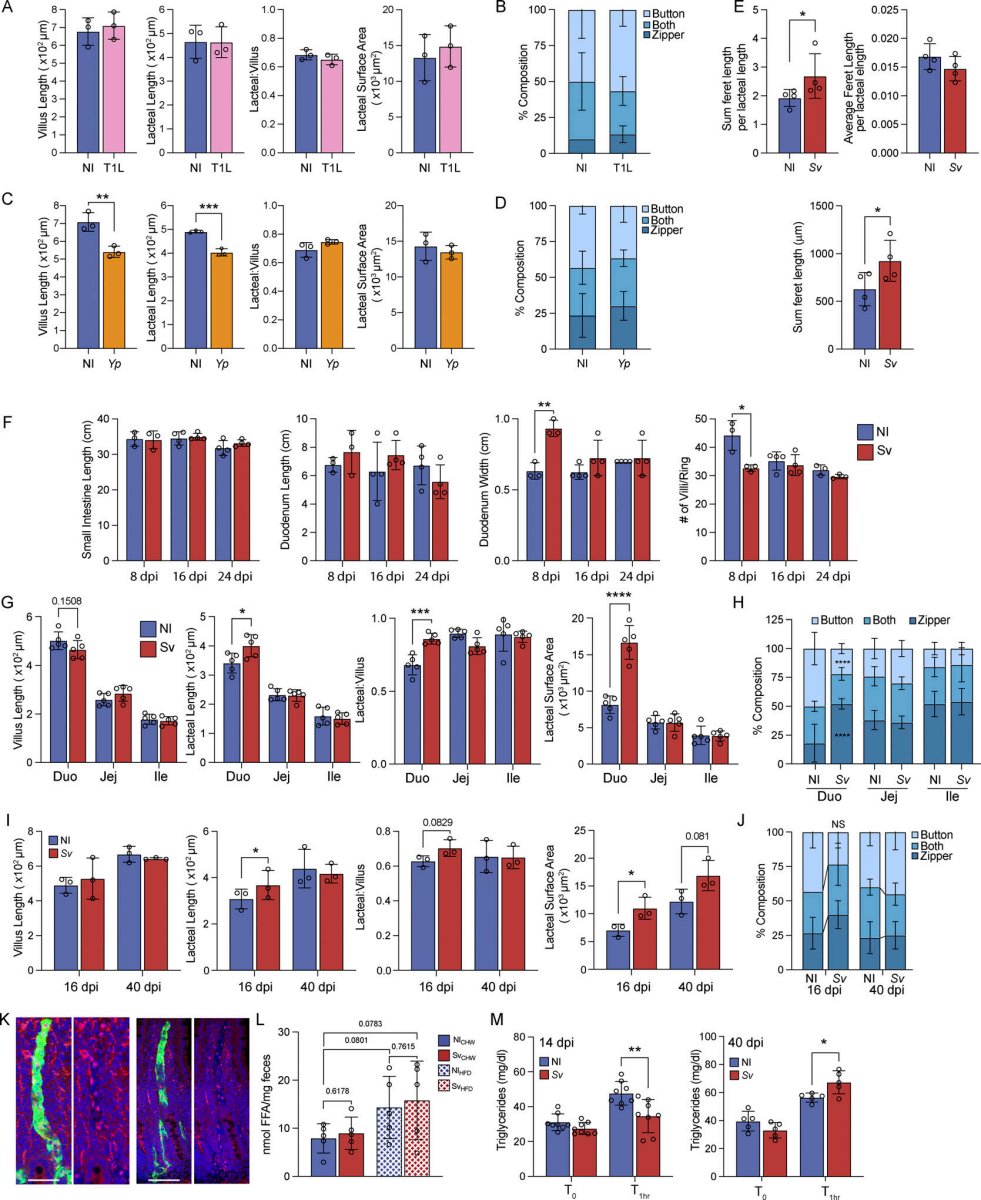
**Helminth infection and not viral or bacterial infection impacts lacteal morphology, only at the site of infection and during active infection** **. (A–D)** Duodenal villus length, lacteal length, lacteal-to-villus length ratio, and lacteal surface area (A and C) or quantification of duodenal lacteal tight junction organization with primarily button-like, zipper-like, or a combination of both tight junction formations throughout the length of the duodenal lacteal (B and D) of mice infected with *Y. pseudotuberculosis* (A and B) or reovirus T1L (C and D) 48 h prior to sacrifice (*n* = 3). Data are representative of one experiment. **(E)** Sum feret/lacteal length, average feret length/lacteal length, and sum feret length per lacteal of mice infected with *Sv* or not (NI) 8 days earlier (*n* = 4). Data are representative of one experiment. **(F)** Small intestinal length and duodenal length, circumference, and villi per diameter of mice infected with *Sv* or not (NI) 8, 16, or 24 days earlier (*n* = 4). Data are representative of one experiment. **(G and H)** Villus length, lacteal length, lacteal-to-villus length ratio, and lacteal surface area (G) and quantification of small intestine lacteal tight junction organization (H) with primarily button-like, zipper-like, or a combination of both tight junction formations throughout the length of the lacteal along the small intestine of mice infected with *S. venezuelensis* (*Sv*) or not (NI) 8 days earlier (*n* = 5). Data are representative of one experiment. **(I and J)** Duodenal villus length, lacteal length, lacteal-to-villus length ratio, and lacteal surface area (I) and quantification of duodenal lacteal tight junction organization (J) with primarily button-like, zipper-like, or a combination of both tight junction formations throughout the length of the lacteal along the small intestine of mice infected with *S. venezuelensis* (*Sv*) or not (NI) 16 or 40 days earlier (*n* = 3). Data are representative of one experiment. **(K)** Representative immunofluorescence images of Prox1 (red)-stained duodenal lacteals (green) in NI and *Sv* mice 8 dpi. Scale bar = 50 μm. **(L)** Fecal FFAs from NI and Sv-infected mice fed a normal chow (CHW) or an HFD for 3 days prior to fecal sampling. **(M)** Serum triglyceride concentration of mice infected with *Sv* or not (NI) 14 or 40 days earlier before and 1 h after gavage with olive oil in the presence of poloxamer (*n* = 7 at 14 days, *n* = 4 at 40 days). Data are representative of two experiments. Error bars indicate the mean ± SD and *P < 0.05, **P < 0.01, ***P < 0.001, ****P < 0.001 by two-tailed Student’s *t* test. Duo, duodenum; Jej, jejunum; Ile, ileum; NI, noninfected.

Given the findings that the duodenum and its associated lymphatic vasculature possess the highest lipid uptake potential, we determined whether infections at this site would impair this lymphatic property. Infections have been shown to be capable of modifying lymphatic function, whereby acute viral skin infection induces rapid dermal lymphatic zippering (Churchill et al., 2022) and bacterial lung infection leads to lymphangiogenesis-associated zippering (Yao et al., 2012). In the gastrointestinal tract, the bacterium *Y. pseudotuberculosis* infects and kills LECs, leading to leakage of migratory cells into the mesentery (Fonseca et al., 2015). We therefore infected adult mice with pathogens that possess either solely duodenal or pan-intestinal tropism and elicit strong immune responses in the draining LNs at the doses used. These include helminths (*Strongyloides venezuelensis* [Esterházy et al., 2019], *Nippostrongylus brasiliensis* [Jenkins, 1974], *Heligmosomoides polygyrus* [Reynolds et al., 2012]), a reovirus strain, T1L (Brown et al., 2023), and *Y. pseudotuberculosis* (Fonseca et al., 2015). At the respective heights of infection, we first analyzed duodenal lacteal morphology and tight junction organization. Each helminth infection led to an increase in the duodenal lacteal-to-villus length ratio and lacteal surface area relative to noninfected controls (Fig. 2, A–F). Lacteal LEC tight junctions switched to a mixed/zipper-like conformation (Fig. 2, G–I). In contrast, reovirus T1L and *Y. pseudotuberculosis* did not change any of these parameters (Fig. S2, A–D). Lacteals were also unaffected in the jejunum of T1L- or *Y. pseudotuberculosis*–infected mice, despite the tropism of these pathogens to this gut segment, or in mice infected with another virus, murine norovirus CW3 (Brown et al., 2023) (data not shown).

To further investigate the effect of helminth infections on lymphatic vasculature, we chose *S. venezuelensis* for further analyses, as it is cleared after 12–14 days, allowing the study of postinfection phenomena, and as it maintains a strict tropism to the duodenum. The increase in zipper-like duodenal lacteal tight junctions was independently confirmed with automated tight junction measurements, indicating that the helminth infection leads to an increase in the sum of tight junction length (Fig. S2 E). Prox1 and Ki67 stainings of the duodenum revealed an increase in the number of Prox1+ nuclei per LYVE-1+ lacteal and an increase in Ki67+ LECs upon *S. venezuelensis* (Fig. 2, J and K; and Fig. S2 K), suggesting that lymphangiogenesis rather than overt cell spread alone underlies the increase in lacteal length and surface area following helminth infection. When examining the whole gut, *S. venezuelensis* infection grossly altered the duodenal tissue, whereby a slight elongation of the duodenum, a significant increase in circumference of the duodenum, and a decrease in the number of villi per intestinal “ring” were observed (Fig. S2 F). Additionally, lacteal scoring of the jejunum and ileum of *S. venezuelensis*-infected mice showed the effects of the helminth infection on the lymphatic vasculature were largely confined to the duodenum, the site of infection (Fig. S2, G and H).

Based on the altered tight junction composition following *S. venezuelensis* infection, we predicted that lacteal dietary lipid uptake might be consequently compromised. To test this, we orally inoculated mice with a bolus of olive oil at the height of infection. Upon *S. venezuelensis* infection, the triglyceride content in the duodenum-draining LNs was substantially decreased; however, it was additionally decreased in the jejunum-draining LN and slightly elevated in the ileum-draining LN (Fig. 2 L). We attribute the jejunal LN effect to the proximal jejunum, which may be affected by local *S. venezuelensis* infection despite a lack of active helminth infection at that site. Correspondingly, plasma triglyceride content decreased in *S. venezuelensis*–infected mice during the 3 h following the oil gavage (Fig. 2 M), while we observed duodenal lipid accumulation (Fig. 2, O and P). These results phenocopy observations made using Vegfr3^ΔProx1^ mice (Fig. 1, G–I) and other models of increased lacteal zippering (Hong et al., 2020; Zhang et al., 2018), further supporting a relationship between tight junction organization and lipid uptake following helminth infection. Finally, we determined the long-term consequence of decreased lymphatic lipid uptake capacity on body weight by placing mice on an HFD or nutritionally matched low-fat diet (60% versus 13% calories from fat) and infecting them with *S. venezuelensis* or not 2 wk after starting on these diets. No difference in weight gain was observed in mice on the 13% diet; however, on the 60% diet, *S. venezuelensis–*infected mice gained less weight at days 8–18 after infection relative to noninfected control animals (Fig. 2 N). We did not monitor the weight beyond this interval, as *S. venezuelensis* is cleared from the intestine around days 12–14 after infection, and lacteal morphology and serum lipid uptake begin to normalize around day 16 after infection (Fig. S2, I, J, and M). Decreased weight gain was unlikely due to decreased food intake, given there was no difference in weight gain when the mice were placed on a normal chow diet where calories come primarily from carbohydrates. Additionally, other studies have reported that helminth infections do not reduce food intake in rodents (Su et al., 2018; Sousa-Ribeiro et al., 2019). Furthermore, primary free fatty acid (FFA) absorption remained intact in infected mice, as fecal FFA levels were low and comparable to noninfected mice (Fig. S2 L).

Collectively, these observations demonstrate that helminth infections alter the structure of duodenal lacteals and compromise duodenal lymphatic lipid uptake, leading to lipid accumulation in epithelial cells and, under HFD conditions, decreased weight gain.

### LECs along the intestine are transcriptionally distinct and duodenal LECs change their transcriptional profile in response to helminth infection

**Figure S3.**
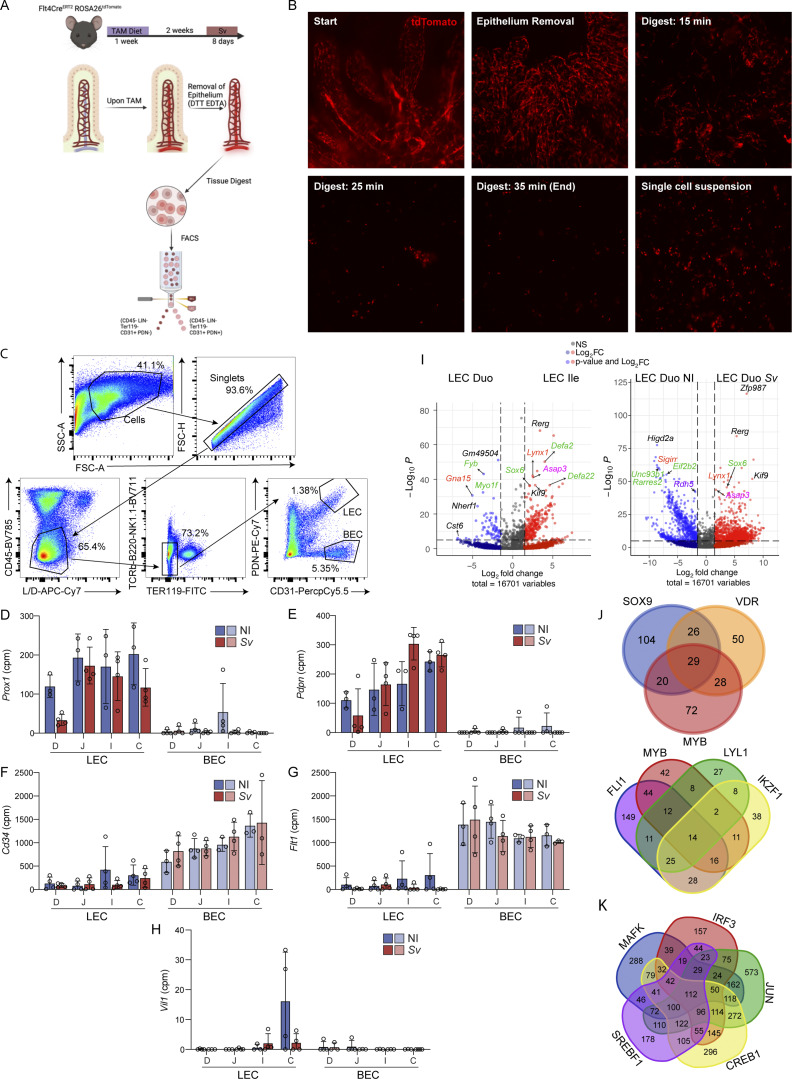
**tdTomato guided intestinal digest, BEC and LEC sorting and bulk RNA sequencing** **. (A)** Schematic of LEC and BEC sorting strategy based on the *Vegfr3*(Flt4)-Cre–mediated expression of tdTomato. **(B)** Immunofluorescence images of digestion state time course of intestine based on Tomato+ cells. At start, Tomato+ cells comprise a lymphatic vessel at the center of each villus and a submucosal blood vessel network. At 15 min into the digestion, some vessels are still intact, and by 35 min into the digestion, a single Tomato+ cell suspension is obtained. **(C)** Gating scheme for sorting BECs and LECs. **(D–H)** cpm for *Prox1* (D), *Pdpn* (E), *Cd34* (F), *Flt1*(G), and *Vil* (H) in sorted LECs and BECs (*n* = 4). **(I)** Volcano plots of DEGs comparing LECs from not infected (NI) Duo and Ile (left) or NI or *S. venezuelensis*–infected Duo and Ile (right). Most highly regulated genes are called out by the gene symbol. Gene symbols in green = immunity-related, orange = environmental sensing, magenta = cell cycle/transcription, purple = metabolism. **(J)** Venn diagrams of the number of genes regulated by indicated TFs accounting for DEGs between NI Duo and Ile. **(K)** Venn diagram of the number of genes regulated by indicated TFs accounting for DEGs between NI or *S. venezuelensis*–infected DUO. D/Duo, duodenum; J, jejunum; I/Ile, ileum; C, colon.

**Figure 3.**
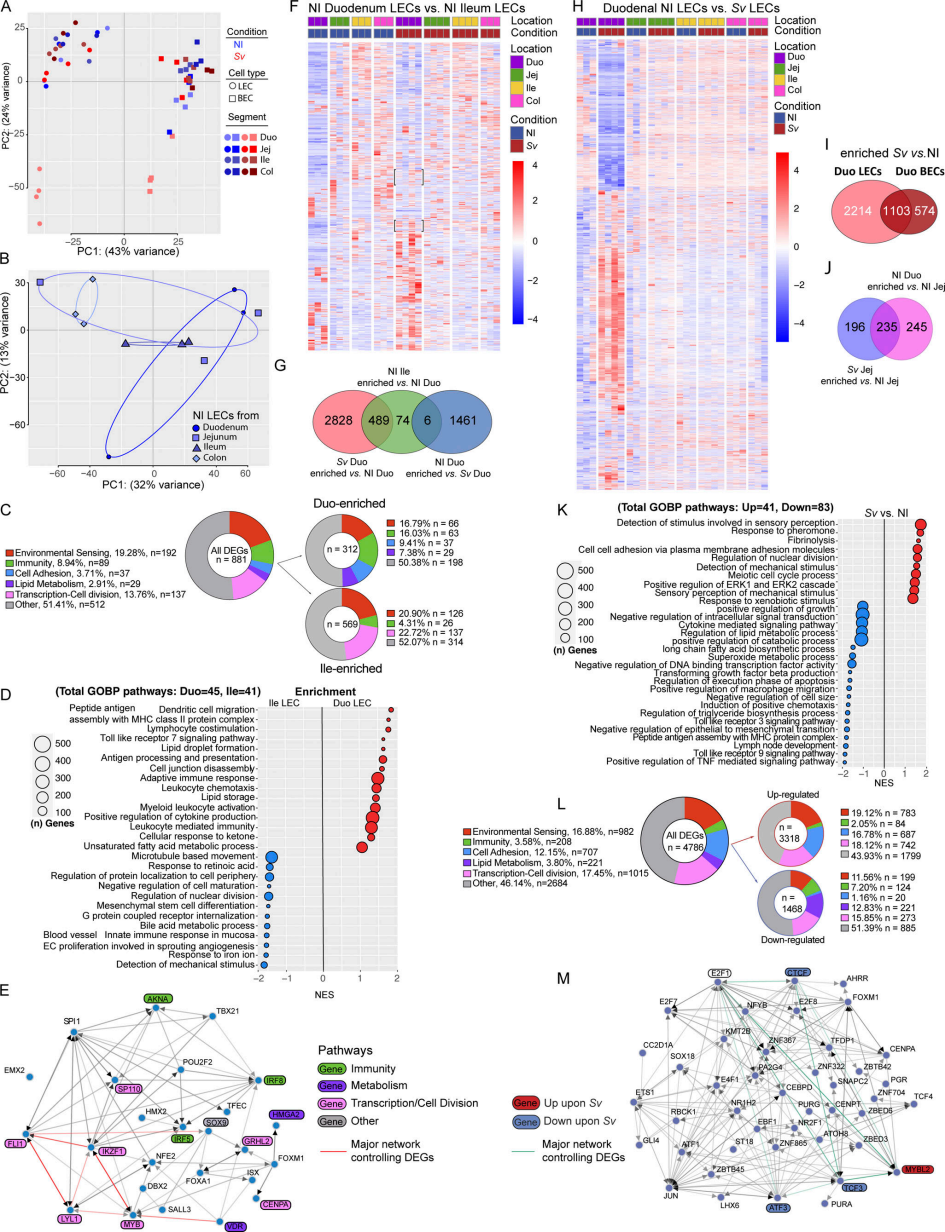
**Duoden** **al** **lymphatics are transcriptionally distinct and respond to helminth infection. (A and B)** Unsupervised PCA of bulk RNAseq of sorted LECs and BECs along the small intestine and proximal colon from NI mice and mice infected with *Sv* 8 days prior (A), or sorted LECs from NI mice (B). **(C–E)** Pie charts depicting the number of genes contributing to general pathways within GOBP pathways of DEGs (C), curated list of GOBP pathway identified by GSEA of DEGs (D), and predicted TF network governing DEGs (E) enriched in LECs from NI ileum or duodenum. TF pathway involvement is color-coded in accordance with C. Major TF interactions regulating DEGs are highlighted. **(F)** Heatmap of DEGs between LECs from NI duodenum and ileum, shown along the intestine, versus upon infection with *Sv* 8 days prior. **(G)** Venn diagram comparing genes upregulated in duodenal LECs upon *Sv* versus LECs from NI mice, genes enriched in ileal LECs compared with duodenal LECs of NI mice, and genes enriched in duodenal LECs from NI mice compared with from mice *Sv* 8 days prior. **(H)** Heatmap of DEGs between duodenal LECs from NI mice versus mice infected with *Sv* 8 days prior, shown along the intestine. **(I)** Venn diagram comparing DEGs in LECs versus BECs sorted from *Sv*-infected duodenum. **(J)** Venn diagram between genes upregulated in jejunal LECs upon *Sv* infection compared with NI mice, and genes enriched in duodenal compared with jejunal LECs in NI mice. **(K–M)** Curated list of GSEA of GOBP into which DEGs (K) and pie charts depicting the number of genes contributing to general pathways within GOBP pathways (L), and predicted TF network governing DEGs (M) between LECs from NI mice and mice infected with *Sv* 8 days prior. TF pathway involvement is color-coded in accordance with L. Major TF interactions regulating DEGs are highlighted. Duo, duodenum; Jej, jejunum; Ile, ileum; Col, colon; GOBP, GO Biological Process; NI, noninfected. *Sv*, *S. venezuelensis*.

We hypothesized that the differences in lacteal structure and lipid uptake capacity along the intestine, as well as alterations in response to helminth infection, would be reflected in the LECs’ gene expression profile. To uncover the molecular underpinnings of these differences and changes upon infection, we performed bulk RNA sequencing (RNAseq) of LECs isolated from the duodenum, jejunum, ileum, and colon from noninfected mice or mice infected with *S. venezuelensis* 8 days earlier. To understand which helminth-induced effects are specific to LECs rather than a general impact on endothelial cells, we expanded this comparison to blood endothelial cells (BECs) (Table S1 A), and we sorted BECs and LECs from Flt4^CreERT2^ x Rosa26^-lsl-Tomato^ mice, using tdTomato to monitor the digestion process (Gardenier et al., 2016; Madisen et al., 2010) (Fig. S3, A–C). In a principal component (PC) analysis (PCA), BECs and LECs of all samples separated by PC1, while PC2 accounted for the duodenal BECs and LECs upon *S. venezuelensis* (Fig. 3 A). We verified our BECs and LECs had minimal contamination by analysis of marker genes, which showed the expected enrichment of *Prox1* and *Pdpn* in LECs, and *Cd34* and *Flt1* in BECs (Fig. S3, D–G); contamination from gut epithelial cells was also minimal, determined by *Vil1* expression (Fig. S3 H).

**Figure 4.**
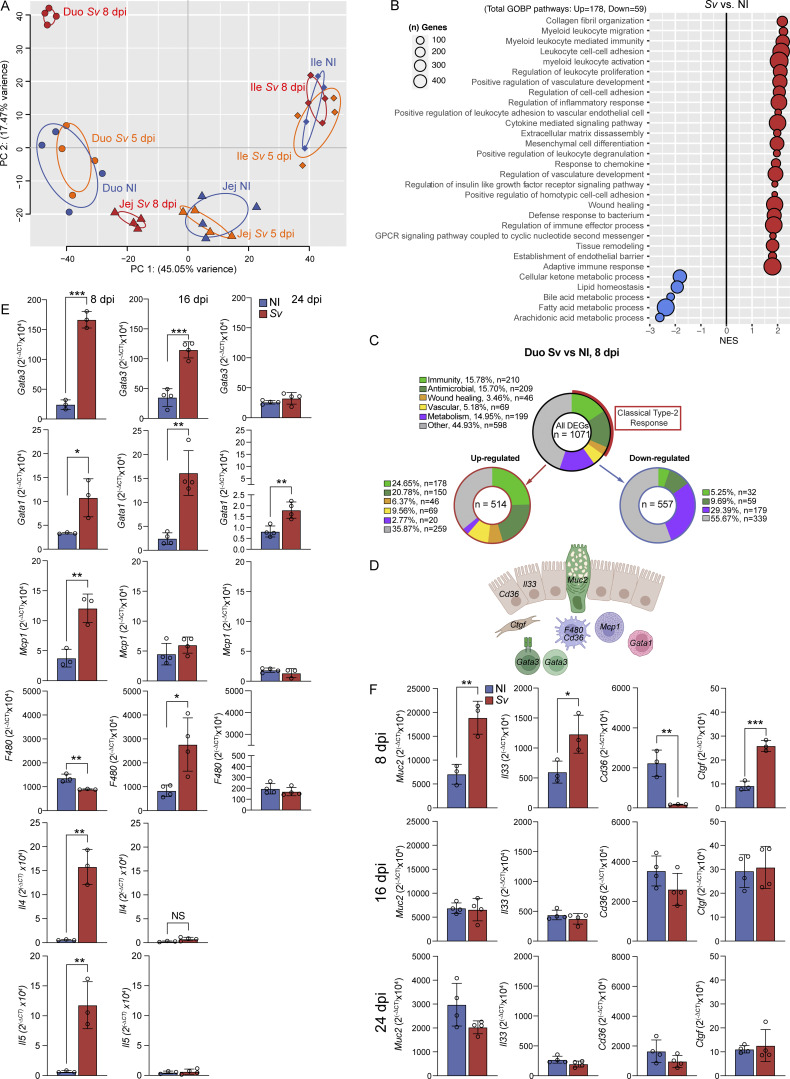
**Mucosal transcriptional signatures in each gut segment are unique and are impacted by helminth infection** **. (A–C)** Bulk RNAseq of mucosal scrapes (without the serosa) along the small intestine from NI mice versus mice infected with *Sv*, 5 and 8 dpi. **(A)** Unsupervised PCA of NI mice and mice infected with *Sv*, 5 and 8 dpi based on cpm. **(B)** Curated list of GOBP pathway identified by GSEA of DEGs between duodenum infected with *Sv* 8 dpi versus NI duodenum. **(C)** Pie charts depicting the number of genes contributing to general pathways within GOBP pathways: all DEGs, upregulated DEGs, and downregulated DEGs. **(D–F)** Canonical anti-helminth type-2 immune effector pathway. **(D)** Schematic of tested genes and population they represent. qPCR of key immune (E)  and non-hematopoietic genes (F) on the whole duodenum of NI mice versus mice infected with *Sv* 8, 16, and 24 dpi (*n* = 3–4). Data are representative of one experiment (16 and 24 dpi). Data are representative of two experiments (8 dpi). Error bars indicate the mean ± SD and *P < 0.05, **P < 0.01, ***P < 0.001 by two-tailed Student’s *t* test. Duo, duodenum; Jej = jejunum; Ile, ileum; GOBP, Gene Ontology Biological Process; NI, noninfected; dpi, days postinfection. *Sv*, *S. venezuelensis*.

**Figure 5.**
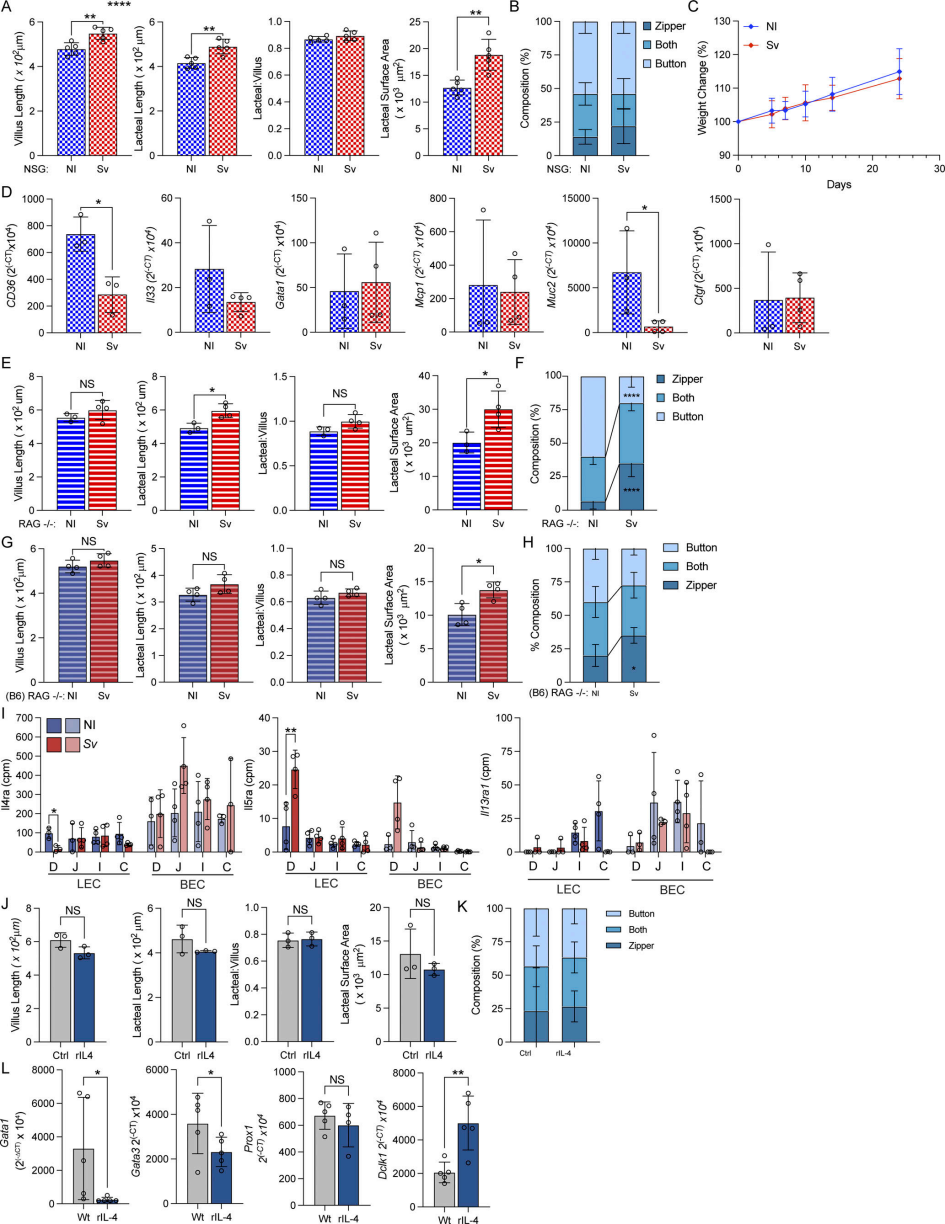
**Innate immunity is necessary and sufficient for *S. venezuelensis*–induced lacteal zippering, while IL-4 alone is not sufficient to trigger lacteal alterations or zippering. (A and B)** Duodenal villus length, lacteal length, lacteal-to-villus length ratio, and lacteal surface area (A) and quantification of duodenal lacteal tight junction organization with primarily button-like, zipper-like, or a combination of both tight junction formations throughout the length of the duodenal lacteal (B) of NSG mice infected with *Sv* 8 days prior (*n* = 5). Data are representative of one experiment. **(C)** Weight change curves of NI C57BL/6J RAG1−/− IL2rg−/− mice infected with *Sv* or not 2 wk after starting a 60% HFD (*n* = 5). Data are representative of one experiment. **(D)** qPCR of key genes involved in the type 2 immune response against helminths from the duodenum of NI and *Sv* NSG mice 8 dpi (*n* = 3–4). Data are representative of two experiments. **(E and F)** Duodenal villus length, lacteal length, lacteal-to-villus length ratio, and lacteal surface area (E) and quantification of duodenal lacteal tight junction organization with primarily button-like, zipper-like, or a combination of both tight junction formations throughout the length of the duodenal lacteal (F) of NOD RAG1–deficient (RAG−/−) mice infected with *Sv* 8 days prior (*n* = 4). Data are representative of one experiment. **(G and H)** Duodenal villus length, lacteal length, lacteal-to-villus length ratio, and lacteal surface area (G) and quantification of duodenal lacteal tight junction organization with primarily button-like, zipper-like, or a combination of both tight junction formations throughout the length of the duodenal lacteal (H) of C57BL/6J *Rag1*–deficient ([B6] RAG−/−) mice infected with *Sv* 8 days prior (*n* = 4). **(I)** Cpm for IL-4, IL-5, and IL-13 cytokine receptors on sorted LECs and BECs from NI mice and mice infected with *Sv* 8 dpi as determined by bulk RNAseq. **(J and K)** Duodenal villus length, lacteal length, lacteal-to-villus length ratio, and lacteal surface area (J) and quantification of duodenal lacteal tight junction organization with primarily button-like, zipper-like, or a combination of both tight junction formations throughout the length of the duodenal lacteal (K) of mice treated with vehicle or rIL-4 (*n* = 5). Data are representative of one experiment. **(L)** qPCR of key genes involved in the type 2 immune response against helminths from the duodenum of mice treated with PBS or rIL-4 (*n* = 5). Data are representative of two experiments. Data are representative of one experiment. Error bars indicate the mean ± SD and *P < 0.05, **P < 0.01, ****P < 0.001 by two-tailed Student’s *t* test. D, duodenum; J, jejunum; I, ileum; C/, colon; NI, noninfected. *Sv*, *S. venezuelensis*; rIL-4, recombinant IL-4 complex.

To determine whether LECs are transcriptionally distinct depending on the segment they drain, we started by comparing the gene signature of LECs from the different intestinal segments of noninfected mice. As expected, the colonic LECs separated from the small intestinal LECs by PCA (Fig. 3 B). Within the small intestine, the duodenal LECs were the most distinct from the other segment LECs, based on the number and nature of differentially expressed genes (DEGs) (Table S1, B–F). The comparison of DEGs between LECs from noninfected duodenum versus ileum revealed that more genes were enriched in the ileum than duodenum (Fig. 3 C). By both gene set enrichment analysis (GSEA) (Fig. 3 C) and overrepresentation gene ontology biological process (GOBP) pathway analysis (Fig. 3 D; and Table S1, G and H), duodenal LECs had a signature indicative of *cell adhesion* and *cell junction disassembly* mechanisms, while ileal LECs were enriched for *active transcription/cell division*. These pathways could reasonably be related to the structural differences observed by immunofluorescence (see Fig. 5 for more detail). Though not directly relevant to answering lymphatic structural attributes, other pathways enriched in duodenal LECs were *immune involvement*, including antigen presentation, and *lipid metabolism*. LECs in both the duodenum and ileum were involved in *environmental sensing*, albeit different pathways (Fig. 3, C and D; and Fig. S3 I). Ileal LECs also featured a bile acid response program (Fig. 3 D). These other pathways indicate that LECs adapt to the milieu in which they sit, which we formally address in Fig. 4. Target-based analysis of the transcription factors (TFs) potentially responsible for this DEG list suggested an intertwined network (Fig. 3 E), of which two groups, the SOX9-VDR-MYB and FLI1-MAFK-IRF3-JUN-SREBF1-CREB1 networks, accounted for more than two thirds of the DEGs (Fig. S3 J). Finally, when comparing the LECs across all segments and conditions collected based on the DEGs between noninfected duodenum and ileum on a heatmap (Fig. 3 F), it became apparent that these DEGs show an intermediate level of expression in the jejunum, suggesting a regulation gradient along the small intestine, while the colon started to resemble the ileum.

Intriguingly, upon *S. venezuelensis* infection, some gene clusters in the duodenal LECs showed resemblance to the ileal patterns (bracketed in Fig. 3 F). Closer analysis of the gene expression profile of duodenal LECs from mice infected with *S. venezuelensis* revealed that of the roughly 3,300 genes upregulated in LECs from infected mice compared with noninfected controls, about 500 overlapped with the genes found enriched in ileal LECs compared with duodenal LECs from noninfected mice (Fig. 3 G). Notably, the overlap consisted of primarily proliferative and developmental genes (Table S1, I and J), suggesting that duodenal LECs acquired the ileal “regenerative” LEC program, as well as a decrease in the lipid metabolism signature upon *S. venezuelensis*. A heatmap of the DEGs between duodenal LECs from noninfected and *S. venezuelensis*–infected mice supported our previous observation based on morphology that the impact of *S. venezuelensis* was restricted to the duodenum (Fig. 3 H). The comparison of the DEGs enriched in duodenal LECs versus BECs upon *S. venezuelensis* indicated that despite the fact that a third of all LEC genes were also regulated in BECs, helminth infection influenced LEC gene expression significantly more than it did the BEC profile (Fig. 3 I and Table S1 K). Jejunal LECs were also somewhat affected by *S. venezuelensis* (~400 DEGs [Fig. 3 J and Table S1 L]), of which about half made the jejunal LECs transcriptionally resemble duodenal LECs (Fig. 3 J), predominantly in their immunity-related signature (Table S1 M). Many DEGs between duodenal LECs from noninfected control versus *S. venezuelensis*–infected mice fell into similar pathways as those when characterizing duodenal versus ileal LECs, including cell adhesion and cell division (Fig. 3, K and L; Fig. S3 I; and Table S1, N and O). The TF network (Fig. 3 M and Fig. S3 K) predicted to regulate the DEGs was dominated by TFs described to be involved in regulating cellular transcription and cell division, and also included TFs known to promote lymphangiogenesis and lymphatic maturation such as *Sox18* and *Jun* (François et al., 2008; Fu et al., 2023).

In sum, RNAseq of LECs along the intestine uncovered that differential permeability properties and diminished lipid exposure along the gut are also reflected transcriptionally, while duodenal LECs upon *S. venezuelensis* feature a proliferative program. These results underscore our structural and functional observations. More globally, the RNAseq analysis suggests LECs adapt to the metabolic and immune milieu in which they are embedded, which change along the gut and in response to helminth infection.

### The mucosal signatures along the small intestine match the functional profiles of LECs, and helminth infection selectively alters the duodenal mucosa

**Figure S4.**
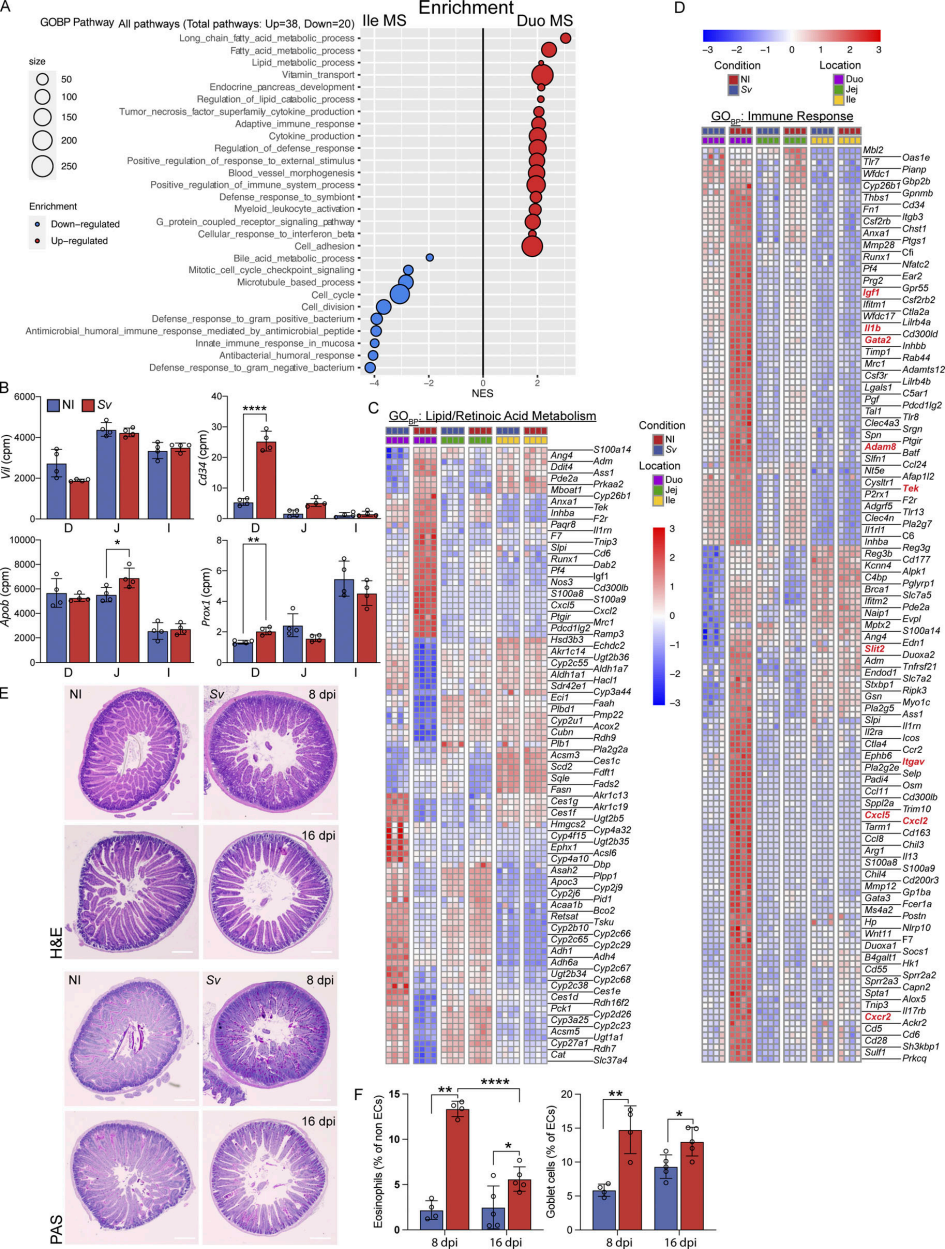
***S. venezuelensis*** **impacts the duodenal mucosa's metabolic and immune-related transcriptional profiles** **. (A)** Curated list of GOBP pathway identified by GSEA of DEGs between mucosal scrape of duodenum versus ileum of NI mice. **(B)** Cpm of epithelial genes (*Vil*, *Apob*), a LEC marker (*Prox1*), and BEC marker (*Cd34*) in mucosal scrapes along the small intestine of NI mice or mice infected with *Sv* 8 days earlier. **(C and D)** Heatmaps of DEGs between duodenal mucosal scrapes of NI mice or mice infected with *Sv* 8 days earlier belonging to the lipid/retinol metabolism (C) or immunity-related (D) GOBP as measured by RNAseq of the mucosal scrapes along the small intestine. **(E and F)** Representative H&E and PAS staining of duodenum tissue from NI and mice infected with *Sv* 8 and 16 dpi (E) and quantification of eosinophil infiltration of non-ECs and goblet cells out of all ECs (F) (*n* = 5). Data are representative of one experiment. Error bars indicate the mean ± SD and *P < 0.05, **P < 0.01, ****P < 0.001 by two-tailed Student’s *t* test. Scale bar = 500 μm. D/Duo, duodenum; J/Jej, jejunum; I/Ile, ileum; GOBP, GO Biological Process; NI, noninfected. *Sv*, *S. venezuelensis*. ECs, epithelial cells.

**Figure 6.**
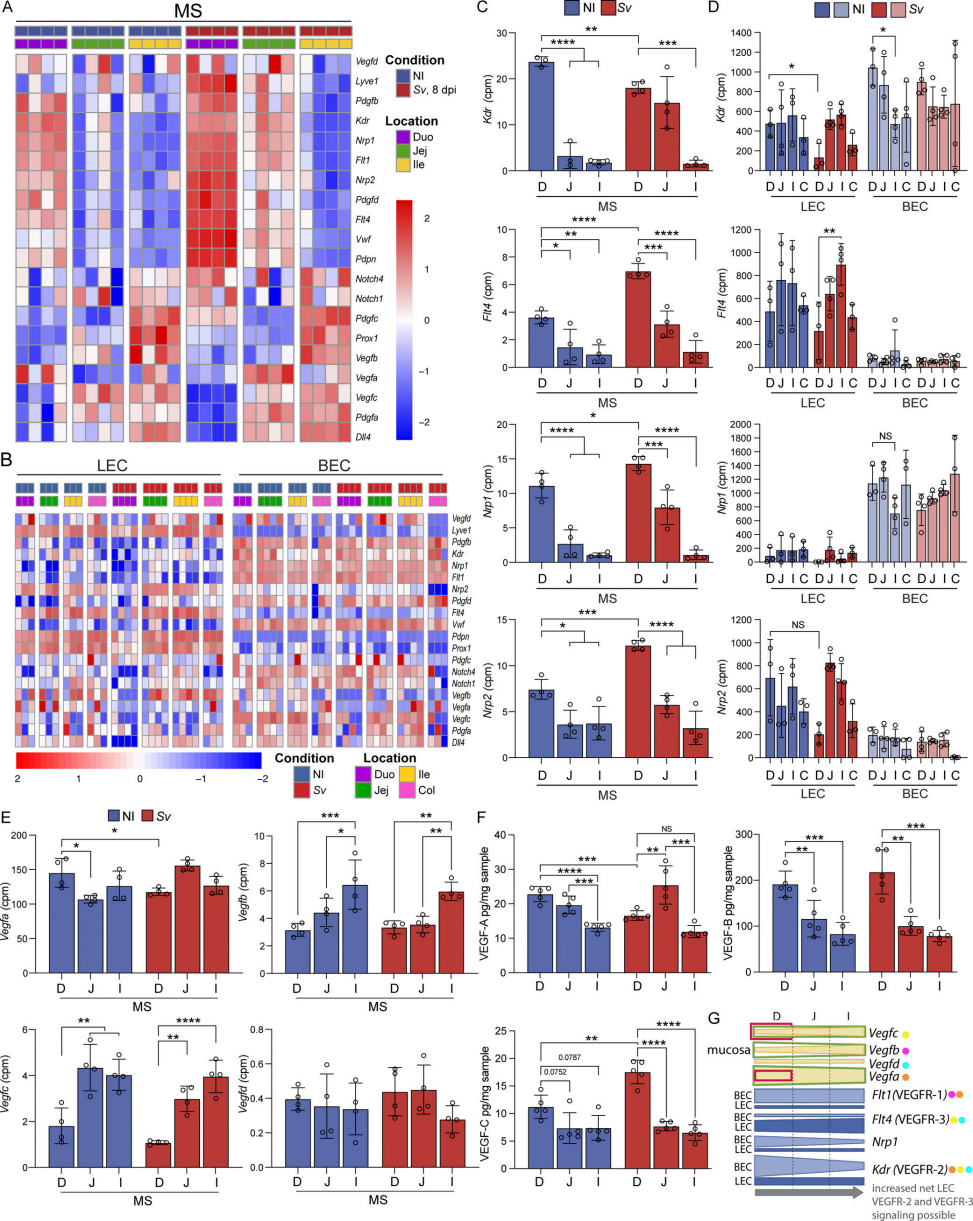
**Canonical BEC and LEC VEGF signaling pathway gene expression correlates with increased lacteal zippering along the small intestine. (A and B)** Expression heatmaps of canonical vascular signaling ligand and receptor signaling pathway molecules relevant to LEC maintenance and remodeling. Mucosal scrape (MS) RNAseq (A) and sorted LEC and BEC RNAseq (B). **(C and D)** Expression in cpm from RNAseq of VEGFRs known to be expressed on LECs along the gut from the mucosal scrape RNAseq (C) and the sorted LEC and BEC RNAseq (D). **(E)** Expression in cpm of VEGFs (*Vegf*) along the gut known to influence LEC behavior from RNAseq on mucosal scrape tissue from NI and *Sv*-infected mice 8 dpi. **(F)** VEGF protein concentrations within each small intestine segment from NI and *Sv*-infected mice 8 dpi (*n* = 5 mice). Data are representative of one experiment. **(G)** Graphic summarizing the mucosal expression (yellow) and protein (green) gradient of VEGFs and expression of their cognate receptors in LECs and BECs (blue). Red boxes in VEGF gradients indicate changes in the protein level upon *Sv* infection. Each VEGF is assigned a color dot, and the order of the dots indicates the ligand preference of the receptors. **(A–F)** Error bars indicate the mean ± SD and *P < 0.05, **P < 0.01, ***P < 0.001, ****P < 0.001 by two-tailed Student’s *t* test (comparison between conditions [C–F]) or one-way ANOVA with Šídák’s multiple comparisons test between single locations (C–F). D/Duo, duodenum; J/Jej, jejunum; I/Ile, ileum; C/Col, colon; NI, noninfected. *Sv*, *S. venezuelensis.*

**Figure 7.**
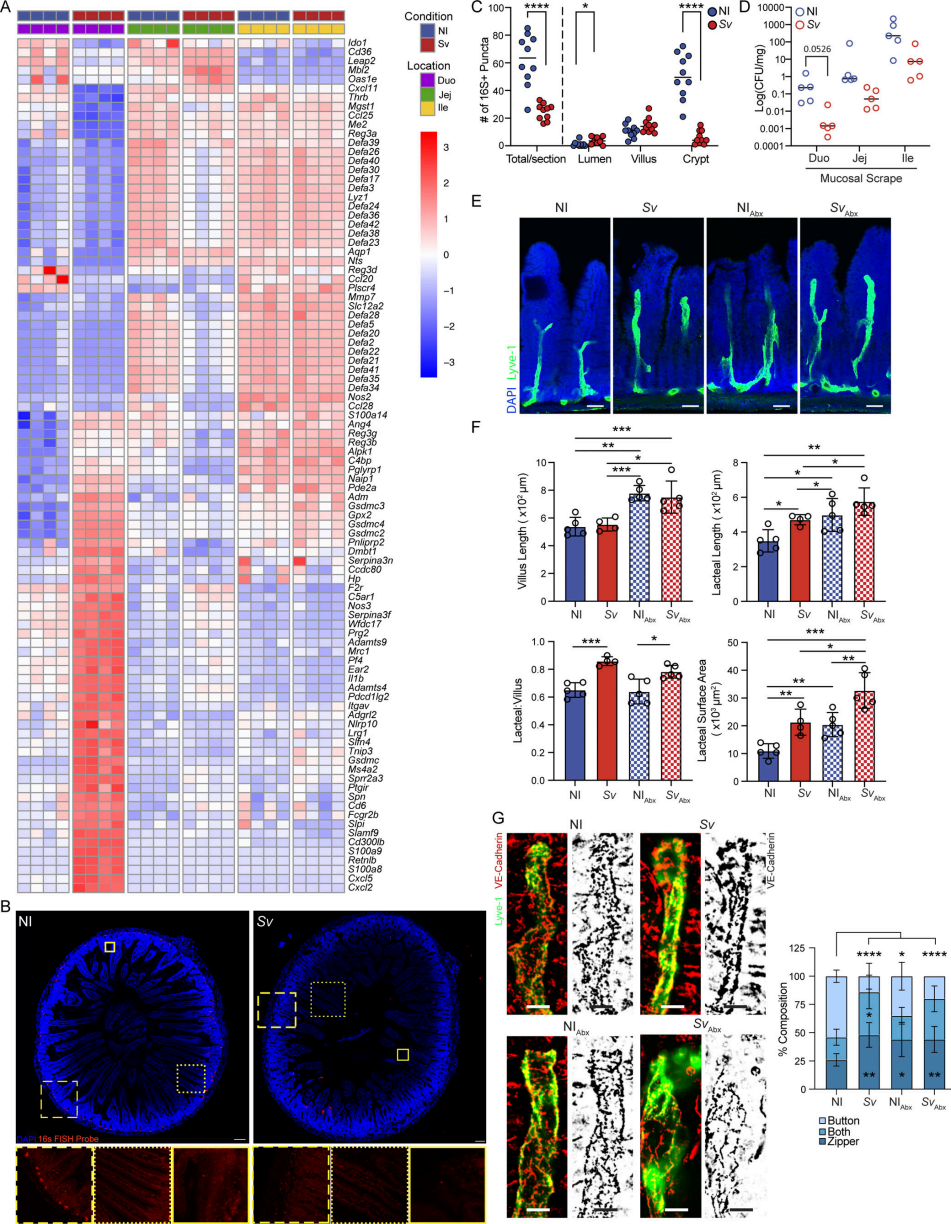
**Microbial depletion does not account for lacteal morphology upon helminth infection. (A)** Heatmap of the expression of antimicrobial pathway genes identified in GOBP pathway analysis in RNAseq of the duodenal mucosal scrape of NI mice versus 8 dpi with *Sv*. **(B and C)** FISH probe (EUB338) detection (B) and enumeration (C) of bacteria in the duodenum of NI mice and mice infected with *Sv*, 8 dpi (dots represent count per duodenum section, two sections per mouse counted, *n* = 5 mice per group). Data are representative of one experiment. Scale bar: 100 μm, zoomed in-picture: 350 μm. **(D)** Microbial CFU within the small intestine mucosal scrape of NI mice and mice infected with *Sv*, 8 dpi, as determined by qPCR. Data are normalized to the sample input weight. (dots represent individual mouse, *n* = 5 mice per group). Data are representative of two independent experiments. **(E and F)** Immunofluorescence images (E) and comparison (F) of duodenum villi and LYVE-1+ lacteals in NI versus *Sv*-infected 8 dpi, NI antibiotic–depleted (NI_Abx_), and Sv-infected antibiotic–depleted (*Sv*_Abx_) mice, 8 dpi: villus length, lacteal length, lacteal length-to-villus length ratio, and lacteal surface area (*n* = 5 mice per group). Data are representative of one experiment. **(G)** Representative images and characterization of VE-cadherin+ LEC junctions of LYVE-1+ lacteals in NI versus *Sv*-infected mice, 8 dpi, NI_Abx_, and *Sv*_Abx_-infected mice, 8 dpi. Quantification of duodenal lacteal tight junction organization with primarily button-like, zipper-like, or a combination of both tight junction formations throughout the length of the lacteal (*n* = 5 mice per group). Data are representative of one experiment. Error bars indicate the mean ± SD and *P < 0.05, **P < 0.01, ***P < 0.001, ****P < 0.001 by two-tailed Student’s *t* test (F) or two-way ANOVA with Šídák’s multiple comparisons test. Scale bar: 100 µm (B and E), 25 µm (G). Duo, duodenum; Jej, jejunum; Ile, ileum; NI, noninfected; dpi, days post infection. *Sv*, *S. venezuelensis.*

To gauge the extent to which the LEC profiles we observed reflected an adaptation to the local milieu both at homeostasis and upon *S. venezuelensis* infection, we performed RNAseq of the duodenal, jejunal, and ileal mucosa. We performed this experiment at 8 days but also 5 days after infection, to investigate the potentially direct regulation of LECs by recently matured worms (Maeda et al., 2019) at a time point at which the host has yet to mount an anti-helminth type 2 immune response in the gut. Unbiased clustering of all samples (Fig. 4 A and Table S2, A–C) showed that the duodenal, jejunal, and ileal mucosae at homeostasis were distinct (PC1), with the genes falling into similar pathways as found enriched in the LECs from the corresponding regions: vascular development, lipid metabolism, immune responses, and cell adhesion were enriched in the duodenum, and cell cycling was enriched in the ileum (Fig. S4 A). These signatures were unlikely to originate from LECs or BECs, which are part of the mucosa, as their contribution to the RNAseq reads was marginal compared with that of the epithelium, based on counts per million (cpm) (Fig. S4 B). Rather, this parallel implies that the LECs are transcriptionally tuned to the local mucosal milieu. The duodenal samples upon *S. venezuelensis* separated on day 8 but not on day 5 after infection from the noninfected mucosa along PC2 (Fig. 4 A), suggesting that the change in duodenal LECs upon helminth infection is not mediated directly by worm-derived molecules. Indeed, when analyzing lacteal morphology at 5 days after infection with *S. venezuelensis*, we observed no change in surface area or tight junctions (data not shown). Also as expected, the ileal mucosa was unaffected by *S. venezuelensis*. The jejunal mucosa on day 8 became more closely related to the noninfected duodenal mucosa (Fig. 4 A), which was due to a similarity in immune-related genes, akin to the observation in the LECs, as well as cell cycle genes (Table S2, D and E). GSEA and an analysis of the number of DEGs within the GOBP pathways into which these DEGs fell (Fig. 4, B and C) uncovered that next to the expected pathways, a part of the classical type 2 response against helminths—inflammatory pathways (Fig. S4 D), antimicrobial responses (Fig. 6 A), and wound healing—pathways related to vascular development, was enriched (Fig. 7 A), while lipid/retinoic acid metabolic process pathways were downregulated (Fig. S4 C).

When comparing the pathway analyses between the duodenal infected mucosa and LECs isolated from the infected tissue, several signatures in the GSEA were congruent, notably cell migration, cell proliferation, and lipid metabolism (compare Fig. 3 K and Fig. 4 B), as was the adoption of more ileum-like profiles (see overlap blue pathways in Fig. 3, D and K [LECs] and Fig. 4 B and Fig. S4 A [mucosa]). Finally, we asked whether the kinetics of the anti-helminth type 2 effector immune cell signature and the nonhematopoietic anti-helminth response reflected the dynamic changes to duodenal lymphatic upon helminth infection at the height of infection and two time points postinfection clearance (early and late) (Annunziato et al., 2015). *Gata3/Il4/Il5*, *Gata1*, *Mcp1*, and *F480* expression was measured by quantitative PCR (qPCR) as proxies for Th2 cells/ILC2s, eosinophils, mast cells, and macrophages, respectively (Fig. 4 D). In the duodenum of mice infected with *S. venezuelensis* or not 8,16, or 24 days before, *Gata3* and *Gata1* still displayed similar elevation at day 16 as day 8 (Fig. 4 E). However, tissue eosinophil counts were lower by day 16 compared with day 8 (Fig. S4, E and F) as determined by H&E staining, *Mcp1* expression and the effector cytokines *Il4* and *Il5* returned to noninfected levels by that time point, and *F480* expression increased upon resolution, likely reflecting the recruitment of tissue repair–associated macrophages. Similarly, genes associated with the epithelial type 2 response (*Muc2* and *Il33*) (Hammad and Lambrecht, 2015), scavenger receptor signaling (*Cd36*) (Hammad and Lambrecht, 2015), and tissue fibrosis and wound repair (*Ctgf*) (Gieseck et al., 2018) were all upregulated by day 8 but returned to noninfected expression levels by day 16 after infection (Fig. 4 F), though goblet cell counts remained somewhat elevated (Fig. S4, E and F). These results imply that changes in lymphatic morphology following infection with *S. venezuelensis* are synchronized with the mounting and resolution of the anti-helminth response.

Overall, these data further support the notion that the LEC profiles in the small intestine are imprinted by the immunological, metabolic, and mitogenic milieu and locally adapt as the environment changes, both along the gastrointestinal tract at homeostasis and upon worm infection.

### Innate immunity is sufficient for *S. venezuelensis***–**induced lacteal zippering, while IL-4 alone is not sufficient to trigger lacteal alterations or zippering

For the remainder of this study, we leveraged our RNAseq datasets to understand the lymphatic permeability and putative regeneration gradient along the small intestine and how *S. venezuelensis* infection reverses it. We first grossly asked if adaptive immune cells and innate lymphoid cells (ILCs) were required, and if type 2 cytokines were sufficient to orchestrate the lacteal phenotypes we observed following *S. venezuelensis* infection. To this end, we initially infected NOD scid γ (NSG) mice or B6 Rag1-Il2rg double knockout mice. Neither of these models clear *S. venezuelensis* for months, and they lack T cells, B cells, and ILCs. While the duodenal lacteal surface area remained increased (Fig. 5 A), lacteal zippering did not occur (Fig. 5 B). Correspondingly, weight gain on an HFD persisted in B6 Rag1-Il2rg double knockout mice, despite infection with *S. venezuelensis* (Fig. 5 C), and most surrogate genes for type 2 inflammation were reversed (Fig. 5 D). In contrast, in Rag1 knockout mice, lacteal zippering still occurred in response to *S. venezuelensis* infection (Fig. 5, E–H). Since type 2 cytokines are anti-lymphangiogenic in the lung (Shin et al., 2015) and in vitro (Savetsky et al., 2015), we addressed what their effect may be in the gut. By far the most abundant receptor in the duodenum was *Il4ra* based on our RNAseq data (Fig. 5 I). We therefore asked if delivery of recombinant IL-4 (rIL-4) for 3 days, the maximum time LECs would have seen the cytokine on day 8 after *S. venezuelensis* infection, was sufficient to change duodenal lymphatic architecture. However, lacteal length, surface area, and tight junction composition were unaltered (Fig. 5, J and K), despite the cytokine having the expected effect on mucosal genes (Schneider et al., 2018) (Fig. 5 L).

**Figure 8.**
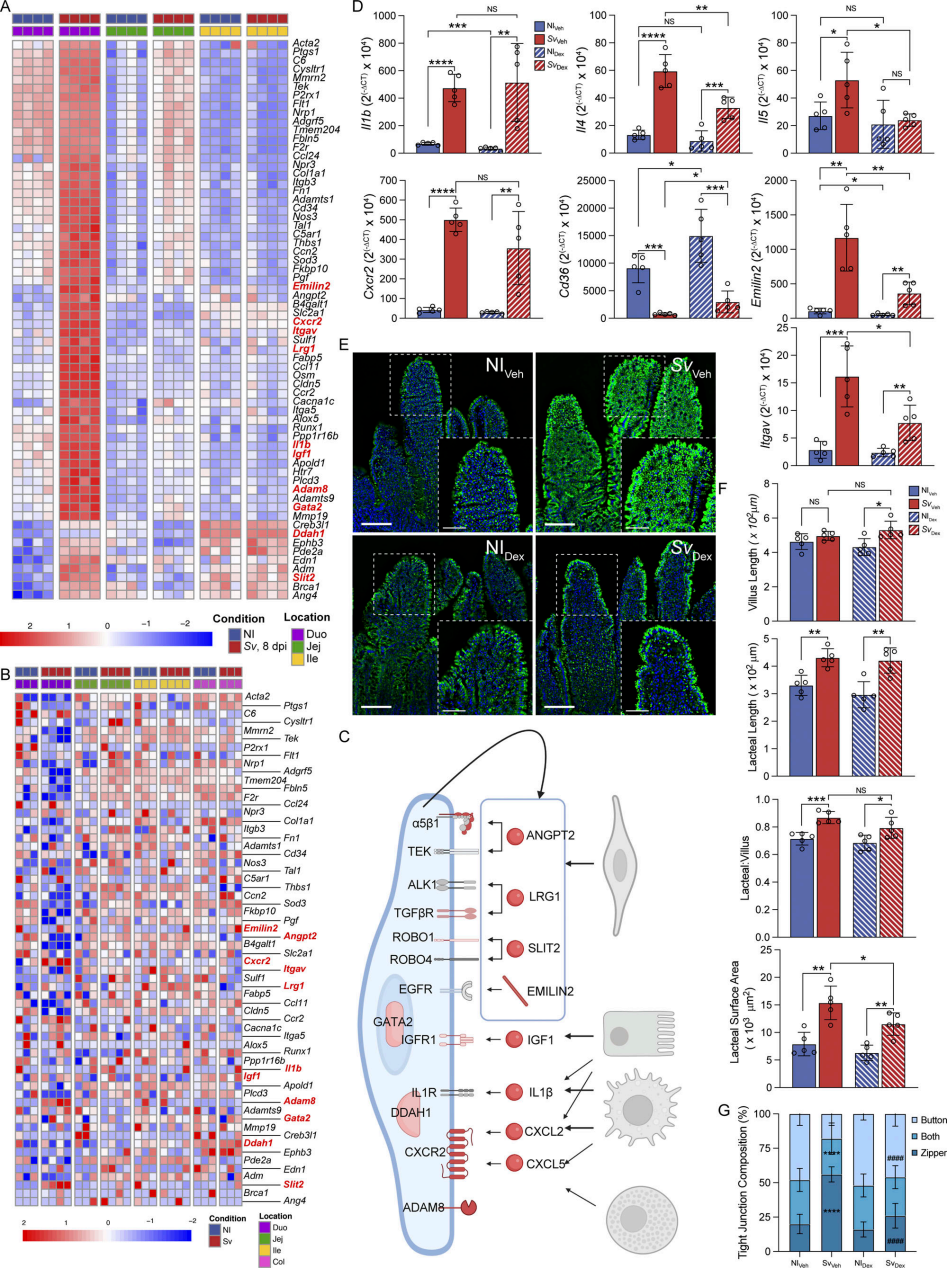
**Inflammation is required for *S. venezuelensis*–induced lymphatic vasculature alterations. (A)** Heatmap of the expression of vascular development–regulatory pathway genes identified in GOBP pathway analysis in RNAseq of the duodenal mucosal scrape (MS) of NI mice and mice infected with *Sv* 8 days prior. **(B)** Heatmap of DEGs between duodenal mucosal scrapes of NI mice or mice infected with *Sv* 8 days prior, belonging to vascular development–regulatory pathway genes as identified by GOBP pathway analysis as measured by RNAseq of the LECs and BECs along the intestine. **(C)** Proposed ligand–receptor interactions between LECs and other cell types within the duodenal microenvironment. Receptors or ligands in red are DEGs in MS or LECs, in pink are trending, in gray are not regulated. **(D)** qPCR of canonical genes involved in the type 2 immune response against helminths within NI mice given vehicle or dexamethasone and mice infected with Sv 8 dpi given vehicle or dexamethasone (*n* = 5). Data are representative of one experiment. **(E)** Representative images of BODIPY staining of duodenal villi from NI_Veh_ and NI_Dex_ mice versus mice infected with Sv 8 dpi treated with either vehicle or dexamethasone after ad libitum feeding. Scale bars are 100 and 50 µm, respectively. **(F)** Comparison of duodenum villi and LYVE-1+ lacteals in NI mice given vehicle (NI_Veh_) alone versus *Sv*-infected mice 8 dpi given vehicle alone (*Sv*_Veh_), NI dexamethasone-treated (NI_Dex_) mice, and *Sv*-infected mice treated with dexamethasone (*Sv*_Dex_), 8 dpi: villus length, lacteal length, lacteal length-to-villus length ratio, and lacteal surface area (*n* = 5 mice per group). Data are representative of one experiment. **(G)** Characterization of VE-cadherin+ LEC junctions of LYVE-1+ lacteals in NI_Veh_ versus *Sv*_Veh_-infected mice, 8 dpi, NI_Dex_, and *Sv*_Dex_-infected mice, 8 dpi. Quantification of duodenal lacteal tight junction organization with primarily button-like, zipper-like, or a combination of both tight junction formations throughout the length of the lacteal (*n* = 5 mice per group). Data are representative of one experiment. Error bars indicate the mean ± SD and *P < 0.05, **P < 0.01, ***P < 0.001, ****P < 0.001 by two-tailed Student’s *t* test (comparison between conditions) (B–J). Duo, duodenum; Jej, jejunum; Ile, ileum; Col, colon; NI, noninfected; dpi, days post infection; *Sv*, *S. venezuelensis*.

Together, these data suggest that Th2 cells are dispensable for the lacteal phenotype as long as ILC2s are present during the anti-helminth response. They also suggest that type 2 cytokines are unlikely to directly act on LECs to elicit structural changes and rather act through indirect mediators, more formally addressed in Fig. 8.

### LEC and BEC VEGFR signaling pathway expression correlates with increased lacteal zippering along the small intestine but not *S. venezuelensis* infection

Lymphatic tight junction organization and regeneration partially intersect on the vascular endothelial growth factor receptor (VEGFR) signaling pathways, and lymphangiogenesis is associated with a zipper-like tight junction conformation that is then reconfigured by the local milieu (Tammela et al., 2007; Yao et al., 2012; Suh et al., 2019). We thus asked whether the expression pattern of VEGF ligands, receptors, and coreceptors could potentially explain our LEC tight junction configurations and proliferative signature both along the gut and upon *S. venezuelensis* infection. The VEGF ligand family consists of four members, VEGF-A, VEGF-B, VEGF-C, and VEGF-D. They bind the receptors VEGFR-1, VEGFR-2, and VEGFR-3 (products of the genes *Flt1*, *Kdr*, and *Flt4*, respectively) with varying affinities, which can lead to crosstalk and modulate the signal strength (Olsson et al., 2006; Simons et al., 2016). Adding further complexity, the expression of receptors on nearby BECs can lead to competition for the ligands (Zhang et al., 2020). On LECs, zipper-like tight junctions are thought to be primarily governed by the net availability of VEGF-A for binding to VEGFR-2 on LECs (Zhang et al., 2020), while VEGF-mediated lymphangiogenesis is carried out by VEGF-C/VEGFR-3 activity (Olsson et al., 2006; Simons et al., 2016).

Analysis of the mucosal scrapes and the sorted LECs and BECs from each gut segment for their expression of genes involved in VEGF signaling alongside LEC marker genes (Fig. 6, A and B) showed that expression gradients exist along the intestine. Most notably, mucosal *Kdr* (VEGFR-2) and *Nrp1*, encoding a VEGFR-2–stabilizing coreceptor, decreased in a proximal-to-distal gradient, in part due to decreased expression by BECs, while LECs maintained equatable expression (Fig. 6, C and D). Mucosal *Flt4* (VEGFR-3) and *Nrp2* also decreased along the small intestine, but their endothelial expression, while strongly enriched in LECs compared with BECs, remained constant (*Nrp2*) or trended to increase (*Flt4*) (Fig. 6, A–D). Similarly, *Flt1* (VEGFR-1) decreased in a proximal-to-distal mucosal gradient but remained the same in isolated endothelial cells and was enriched in BECs compared with LECs, suggesting the mucosal gradients were due to total endothelial cell number gradients. With regard to VEGFR ligand expression, *Vegfa* was the dominant VEGF expressed in the gut (Fig. 6 E, highest cpm) and was downregulated in the jejunum; however, the expression of *Vegfc* and *Vegfb* was twice as high in the jejunal and ileal mucosa compared with that of the duodenum (Fig. 6 E). On the protein level, these gradients held true for VEGF-A; however, VEGF-B displayed an inverse gradient to its transcript count and VEGF-C was not significantly changed along the gut in the noninfected setting (Fig. 6 F). We were unable to recover sufficient isolated endothelial cells from the gut tissue to quantify receptor density within BECs and LECs on a protein level. However, we hypothesize the following: as sequestration of VEGF-A by VEGFR-2 on BEC decreases along the small intestine, free VEGF-A is available for LEC VEGFR-2 binding, permitting more VEGFR-2 activity on LECs, leading to increased lacteal zippering in the distal small intestine. In the ileum, VEGF-C is likelier to promote lymphangiogenesis and zippering associated with this process, rather than VEGFR-2–mediated zippering (Fig. 6 G). Overall VEGFR expression increased in the duodenal mucosa upon *S. venezuelensis* infection (Fig. 6, A and C); however, this was likely due to the increase in LEC and BEC numbers following infection, as on a per cell basis, the receptors on LECs remained the same or decreased (Fig. 6, B and D).

In *S. venezuelensis*–infected mice, *Vegfa* and VEGF-A protein were decreased in the duodenum (Fig. 6, E and F); however, the VEGF-C protein increased upon infection, despite *Vegfc* transcriptionally decreasing (Fig. 6 F), suggesting that the growth factor may be posttranscriptionally regulated. Overall, these analyses put forward a putative VEGFR signaling gradient that may underlie the ascending lymphatic tight junction and LEC proliferative gradient along the small intestine under homeostatic conditions. Elevated VEGF-C upon *S. venezuelensis* infection is a plausible effector mediating the change in lymphatic vasculature morphology and proliferative transcriptional state (Fig. 6 G).

### Mucosal microbial depletion does not account for the change in lacteal morphology upon helminth infection

**Figure S5.**
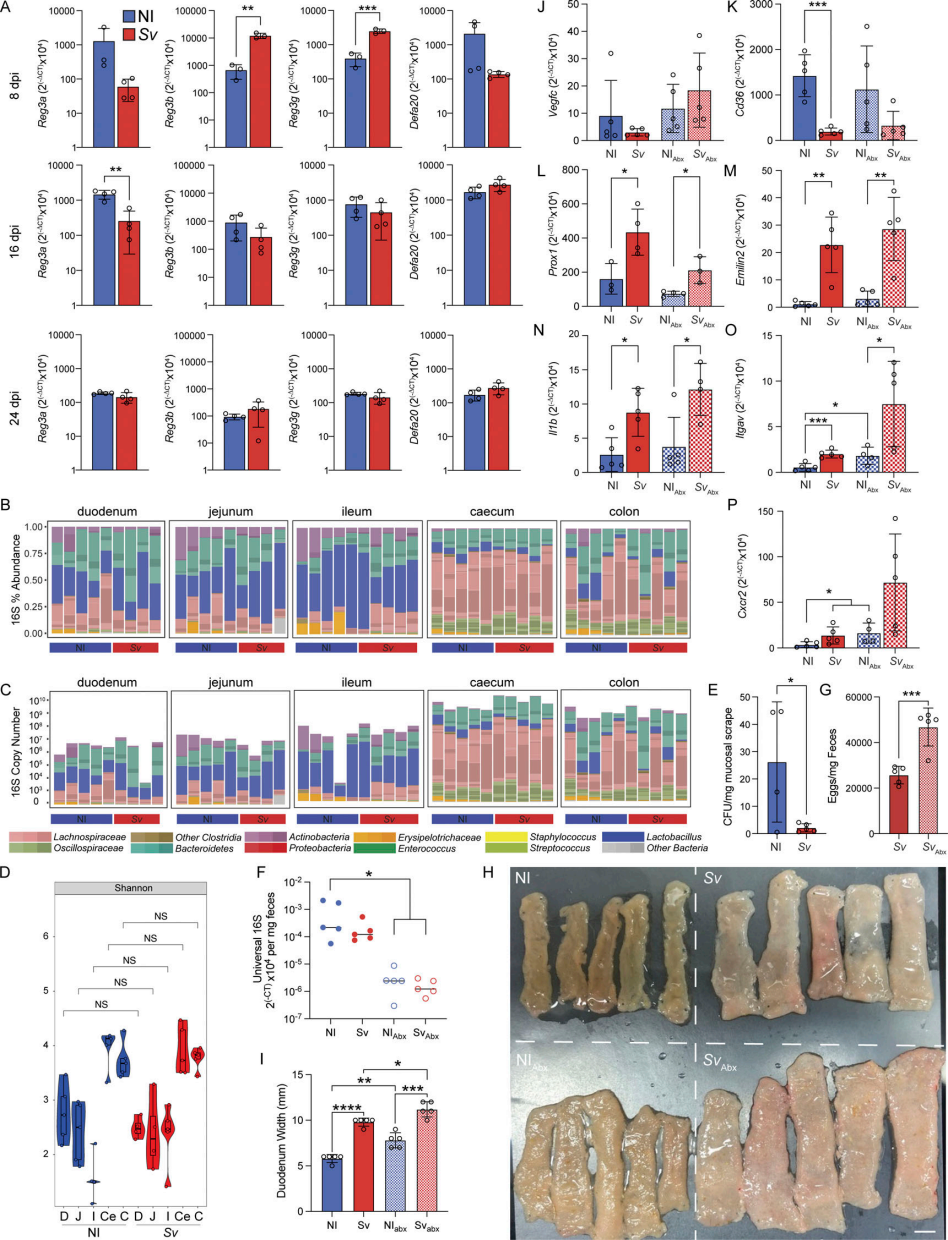
***S. venezuelensis*** **infection impacts duodenal antimicrobial gene expression and tissue morphology but not luminal microbiota composition and load** **. (A)** qPCR for *Reg3a*, *Reg3b*, *Reg3d*, and *Defa20* in whole duodenum of age-matched NI mice or 8, 16, or 24 days post-*Sv* infection (dpi) (*n* = 3, 4, and 4 for 8, 16, and 24 dpi, respectively). **(B–D)** Relative abundance (B), absolute abundance (C), and diversity (D) of bacterial phyla based on 16S sequencing of luminal contents along the gut of NI mice or mice infected with *Sv* 8 days earlier. **(E)** CFUs in duodenal mucosal scrapes of NI mice or mice infected with *Sv* 8 days earlier (*n* = 5). Data are representative of one experiment. **(F)** qPCR for pan-bacterial ribosomal 16S gene in feces of mice treated with broad-spectrum antibiotic cocktail (Abx) or not, and infected with *Sv* 8 days earlier or not (*n* = 5). Data are representative of one experiment. **(G)** Fecal egg count of mice infected with *Sv* and treated with Abx or not (*n* = 5). Data are representative of one experiment. **(H and I)** Picture of longitudinally opened duodena (H) and duodenal diameter (I) of mice treated with broad-spectrum antibiotic cocktail (ABx) or not, and infected with *Sv* 8 days earlier or not (*n* = 5). Data are representative of one experiment. Scale bar = 5 mm. **(J–P)** qPCR of the indicated gene in extracts from whole duodenum of mice treated with broad-spectrum antibiotic cocktail (ABx) or not, and infected with *Sv* 8 days earlier or not (*n* = 5). Data are representative of one experiment. Error bars indicate the mean ± SD and *P < 0.05, **P < 0.01, ***P < 0.001, ****P < 0.001 by two-tailed Student’s *t* test. D, duodenum; J, jejunum; I, ileum; C, colon; dpi, days post infection. *Sv*, *S. venezuelensis*.

The intestinal microbiota has previously been proposed to alter lacteal morphology, as microbial depletion was found to induce lacteal zippering via VEGF-C (Suh et al., 2019). We therefore asked whether the *S. venezuelensis*–induced antimicrobial response (Fig. 4 D, Fig. 7 A, and Fig. S5 A), via microbial depletion, was responsible for the zipper-like duodenal tight junctions observed upon helminth infection. Notably, others have described an antimicrobial response after *H. polygyrus* infection (Hu et al., 2021), suggesting this is a common reaction to helminths. We first interrogated if the observed antimicrobial signature at 8 days after infection with *S. venezuelensis* correlated with an impact on microbial diversity and abundance in our facility (Afrin et al., 2019). However, luminal microbial composition and load were largely unaffected by *S. venezuelensis* infection (Fig. S5, B–D). Given the antimicrobial response is spatially localized in the mucosa, we next assessed whether the tissue-associated microbiota was affected by *S. venezuelensis.* Fluorescence in situ hybridization (FISH) detection of the microbiota with universal 16S DNA probes on the duodenum upon *S. venezuelensis* exposed a decrease in probe signal at the intestinal crypts (Fig. 7, B and C). This decrease in microbial abundance was independently confirmed via a qPCR-based method to quantify bacterial colony-forming units (CFUs) in the mucosa (Fig. 7 D), and via a decrease in CFUs from plated infected mucosal scrapes compared with scrapes from noninfected mice (Fig. S5 E).

To determine whether microbial depletion induces lymphatic changes, we next put mice on broad-spectrum antibiotics (Abx) 3 days prior to and during *S. venezuelensis* infection and then assessed duodenal lacteal morphology 9 days after infection. Successful microbial depletion was confirmed by 16S qPCR (Fig. S5 F). Abx did not compromise worm fecundity and rather led to an increased egg load at 9 days after infection (Fig. S5 G). In some respects the effects of antibiotic-mediated depletion and *S. venezuelensis* appeared additive: lacteal length and surface area (Fig. 7, E and F), as well as duodenal width (Fig. S5, H and I), increased upon either perturbation alone but were further increased when antibiotic-treated mice were infected with the helminth. By contrast and in line with observations by others (Suh et al., 2019), duodenal *Vegfc* expression was not affected by antibiotics (Fig. S5 J). Also, the expression of genes identified to be regulated by *S. venezuelensis* and potentially responsible for the change in LEC morphology and junction composition was either unaffected by antibiotic treatment (Fig. S5, J–M) or behaved additively (Fig. S5, O and P). The only effect of Abx that was not additive with that of *S. venezuelensis* infection was duodenal lymphatic zippering (Fig. 7 G), probably because the maximal zippering possible is achieved with either intervention alone. These results suggest that antibiotics and *S. venezuelensis* infection act on duodenal lymphatics in independent ways.

In sum, these data exclude *S. venezuelensis*–induced mucosal microbial depletion as the primary mechanism through which helminths alter duodenal lacteals.

### Inflammation is required for *S. venezuelensis***–**induced lymphatic vasculature alterations

We finally turned to the lymphangiogenic signatures observed in the LEC RNAseq data as a mechanism underlying *S. venezuelensis*–induced changes in lymphatic architecture and zippering. While tight junction organization and lymphangiogenesis each converge onto VEGF signaling, the processes can be modulated by other, potentially upstream, pathways (Olsson et al., 2006; Simons et al., 2016; Zheng et al., 2014a; Tammela and Alitalo, 2010).

Based on the genes contributing to the vasculature and cell cycling signature pathways upon *S. venezuelensis* in the duodenal mucosa RNAseq (Fig. 8 A), we asked whether any of these genes were also upregulated in isolated LECs upon helminth infection. *Slit2*, *Cxcr2*, and *Adam8* displayed a significant upregulation, and a few more trended (*Gata2*, *Itgav*, *Ddah1*) in the duodenal LECs of *S. venezuelensis*–infected mice (Fig. 8 B). Several secreted factors that may act on LECs without a change in receptors and have previously been found to be linked to LEC maintenance and development (*Il1b*, *Igf1*, *Angpt2*, *Emilin2*, and *Slit2*) were upregulated in the RNAseq datasets (Baluk et al., 2013; Li et al., 2013; Zheng et al., 2014b; Gale et al., 2002; Colombatti et al., 2012; Yang et al., 2010) (Fig. 8, A and B). A significant fraction of these genes also appeared in the immune response pathway (Fig. S4). Based on literature search (Farnsworth et al., 2019; Baluk et al., 2013; Li et al., 2013; Zheng et al., 2014b; Gale et al., 2002; Colombatti et al., 2012; Yang et al., 2010) and ImmGen (for immune cell sources), putative sources for the secreted factors ranged from macrophages, mast cells and enterocytes to BECs and LECs themselves (Fig. 8 C).

While the resulting receptor–ligand pairs are likely not exhaustive (Fig. 8 C), these analyses suggested that inflammation-induced lymphangiogenesis and associated zippering may be responsible for the lymphatic changes after helminth infection. To directly address if this was the case, we treated mice with the anti-inflammatory agent dexamethasone or vehicle (days 4–7 after infection) and asked what the impact on day 8 was on the duodenal lacteal phenotypes induced by *S. venezuelensis*. Of note, previously dexamethasone has been shown to reverse lymphangiogenesis-induced zippering in the lung after bacterial infection (Yao et al., 2012). Transcriptionally, dexamethasone partially reversed the induction of several surrogate genes in duodenal extracts, notably *Il4*, *Il5*, *Cd36*, *Emilin2*, *Itgav*, with only *Il1b* and *Cxcr2* remaining as upregulated as in vehicle-treated mice (Fig. 8 D). This was not due to a milder infection, as egg counts were the same between vehicle- and dexamethasone-treated mice (data not shown). Dexamethasone also reversed the epithelial lipid accumulation (Fig. 8 E), which went in hand with a partial reversal of the increased lacteal surface area (Fig. 8 F) and a near-complete normalization of the duodenal lacteal tight junction distribution (Fig. 8 G). These data support the conclusion that inflammation-induced zippering is the mechanism by which helminths alter lacteal permeability in the duodenum.

Taken together, these results suggest that *S. venezuelensis* triggers lymphangiogenesis and the zippering associated with this process through inflammation. While most likely type 2 cytokines orchestrate this in the case of helminth infection, the data indicate that it is the downstream inflammatory response rather than these cytokines directly that act on LECs.

## Discussion

In this study, we uncovered regional transcriptional and permeability properties of the small intestinal lymphatic vasculature that match the mucosal milieu that they drain. We also highlight how these lymphatics can respond to inflammatory insults, such as a helminth infection, then return to steady-state function once the pathogen is cleared. These findings enhance a mechanistic understanding of the dynamic nature of intestinal lymphatics in health and disease.

Duodenal lymphatics are uniquely poised to maximize lipid uptake. This may be initially substrate-driven during early postnatal development (Zarkada et al., 2023), but once established, this is a structurally stable feature at homeostasis (see Fig. S1, I and J). The phenomenon spans the three-dimensional architecture, lacteal LEC tight junction organization (primarily button-like compared with the rest of the small intestine), and the transcriptome, which indicated fat and retinol utilization by duodenal LECs. How these permeability and metabolic properties synergize with other unique features of duodenal LECs such as their immunostimulatory profile and properties will be interesting to examine in the future, especially since fatty acid use and involvement in immunity are extensively studied and core features of the lymphatics at other sites (Wong et al., 2017; Montenegro-Navarro et al., 2023; Humbert et al., 2017). Similarly, since lymphatic lipid uptake also increases lymph flow rate (Turner and Barrowman, 1977), it will be important to determine how this duodenal uptake bias translates into a flow rate gradient along the intestine and may influence immune outcomes in the draining LNs. The low microbial load in the duodenum due to active immune surveillance may also influence the LEC profile. It is likely that the microbiota functions in shaping the more distal intestinal LEC transcriptome.

Functionally, we focused on the phenomenon of lymphatic permeability and its role in chylomicron uptake. Our data suggest that the molecular basis of this process under homeostatic conditions, likely attributable to the balance of VEGF signaling on LECs versus BECs along the small intestine, is not the same as under inflammatory conditions, where lymphangiogenesis-associated zippering transiently dictates permeability. Lymphangiogenesis also appears to be governed by different triggers under inflammatory conditions than during development or homeostatic turnover. It is possible that inflammation influences both tight junction composition and perceived increased lacteal surface area through additional mechanisms, such as tissue edema, which will raise lymphatic fluid pressure. To this point, a recent study of dermal lymphatic tight junctions demonstrated that imposing mechanical stretch in 2D LEC cultures (mimicking an increase in intravessel pressure or lymph volume) affects tight junction appearance, leading to a more zipper-like phenotype (Schoofs et al., 2025). Notably, in the small intestine, inflammation can not only be triggered by acute infections but is also occurring during chronic conditions such as Crohn’s disease or untreated celiac disease, both of which are associated with nutrient malabsorption.

Transcriptional profiling of LECs along the intestine has been a long-standing gap in knowledge. Our study fills this gap and uncovered compelling regional differences, even within the small intestine, but likely only scratches the surface, given that we bulk-sorted LECs. With improved spatial transcriptomics on the horizon, permitting single-cell resolution and high transcriptome coverage, the current hurdles of yield and cell death of endothelial and rare cells in the gut will soon be surmountable. Such approaches will also give more insights into the potential regional differences of the stromal cells supporting the lymphatics and can be more easily expanded to human tissues.

Helminths turned out to have the strongest effect on the duodenal lymphatics among the infectious agents tested. This observation may have been due to timing, but it more likely depends on the nature and degree of damage and corresponding response that these pathogens elicit compared with the viruses and bacteria we studied. Whether other type 2 immune response–eliciting pathogens or type 17–inducing pathogens cause a similar lymphatic phenotype in the duodenum remains to be determined. However, since dampening inflammation alone reverses the lymphatic phenotype, the prediction of our finding is that any insult that triggers a repair-associated (lymph)angiogenic response will precipitate similar effects. In the duodenum, compromised lymphatic function causes lipid malabsorption; injury at other sites may have different consequences. Resistance to weight gain following gastrointestinal helminth infection despite being fed an HFD has been attributed to peripheral metabolic effects such as altered adipose tissue and distal macrophage behavior (Kabat et al., 2022; Kabat et al., 2023; Su et al., 2018) rather than studying the gut directly. Our data strongly suggest that helminths already influence efficient fat absorption in the intestine. While we monitored triglycerides because of its ease, this phenomenon likely means that many types of molecules packaged into chylomicrons, such as fat-soluble vitamins, are also less efficiently absorbed.

The focus of our study was the intestinal lymphatic vasculature, but we also observed lipid stalling in the epithelial cells as a consequence of lymphatic dysfunction. The epithelium seems to respond to this overload by partially downregulating importers, but still, this likely imposes stress on epithelial cells. On the other hand, the exclusion of the local mucosal microbiota due to worm infection could have beneficial effects in conditions of preexisting barrier breach. From this standpoint, the currently discussed potential uses of helminths as a weight loss measure and to counter inflammatory bowel diseases should be evaluated with the distinct therapeutic goals and benefits in mind, and particular attention paid to the gut segment that the worm of choice infects.

### Limitations of experimental approaches

RNAseq does not capture difference occurring on the translational, protein trafficking, secretion, and signaling level. In LECs, these aspects are hard to capture in vivo without yet to be made tools such as molecular sensors. Our LEC RNAseq experiments are based on a single time point post infection. Therefore, we did not determine whether the structural normalization of the lymphatics after worm clearance occurred with the same kinetics as a transcriptional return to baseline of LECs. Finally, while *S. venezuelensis* is a useful model for studying lymphatic plasticity, it may not capture how lymphatics adapt to chronic helminth infections or sustained inflammation.

## Materials and methods

### Animals

All mouse studies were approved by the University of Chicago Institutional Animal Care and Use Committee. Mice were maintained at the University of Chicago animal facilities under MNV-, SFB-, *Hpp*-free specific pathogen–free (SPF) conditions. B6 (C57BL/6J), B6.129S7-*Rag1*^*Tm1mom*^/J and NOD.129S7(B6)-*Rag1*^*tm1Mom*^/J, NOD.Cg-*Prkdc*^*scid*^*Il2rg*^*tm1Wjl*^/SzJ (NSG mice), B6.129S-*Rag2*^*tm1Fwa*^*Il2rg*^*tm1Wjl*^/J, and TdTomato (B6.Cg-Gt(ROSA)26Sor^tm14(CAG-tdTomato)Hze^/J) were purchased from the Jackson Laboratories and maintained in-house. Flt4^creERT2^ (Flt4^tm2.1(cre/ERT2)Sgo^) (Gardenier et al., 2016) mice were obtained from Dr. Daniel Mucida (The Rockefeller University) with consent of Dr. Sagrario Ortega (National Cancer Research Center, Spain), Vegfr3^lox/lox^ mice were obtained from Dr. Melody A. Swartz (University of Chicago) after material transfer agreement with Dr. Kari Alitalo (University of Helsinki), and Prox1^creERT2^ (Jannaway et al., 2023) mice were obtained from Dr. Joshua P. Scallan (University of South Florida, after material transfer agreement), all maintained in-house following rederivation into our facility in order to achieve consistent SPF status. In general, a mix of male and female mice was used for this study, and animals were 7–10 wk of age. Exclusively male mice were used for all experiments involving helminth infections due to the relative resistance of female mice to helminths. Unless otherwise indicated, mice were on the C57BL/6J background and littermates were used as controls.

### Blocking antibody

VEGFR-2 blocking antibody (DC101, BP0060; Bio X Cell; 45 μg/g) and isotype control antibody (eBR2a, 16-4321-85; Thermo Fisher Scientific; 45 μg/g) were injected intraperitoneally once every 24 h for 96 and 48 h for the *S. venezuelensis* and triglyceride challenge experiments, respectively (all diluted in sterile PBS).

### Dietary interventions

To induce Cre activity, mice were placed on a diet containing 500 mg/kg tamoxifen (TD.130857; Inotiv) for 1 wk and taken off the diet for 1 wk before further interventions.

To study the effect of omission of dietary fat on lymphatic morphology, 6-wk-old C57BL/6J mice were placed on a chow diet (2020x; Inotiv, 16% of calories from fat) or the same diet without added soybean oil (TD.180890; Inotiv, 0.5% of calories from fat, which come from the grain shells) and sacrificed 4 wk later in the morning. For an overnight fast, one group of mice fed diet 2020x for 4 wk had its food removed and cages exchanged for 16 h prior to sacrifice the following morning. The cages of all mice to be compared were exchanged the late afternoon before sacrifice. Rag ko IL2rg−/− mice were put on 60% calories from fat diets at age 11 wk and infected with *S. venezuelensis* 10 days later. Body weights were recorded weekly. For studying the interaction between helminth infection and increased dietary fat content, 6-wk-old male B6 mice were placed on either a refined diet with 13% calories from fat (TD.200502; Inotiv) or a diet with 60% calories from fat (TD.200501; Inotiv) that was otherwise isonutritional to the 13% fat content diet. Mice were fed these diets for 2 wk prior to infecting mice with *S. venezuelensis* and then kept on the diet during the infection. Food was refreshed every week throughout the experiment. Mice were weighed prior to being put onto the diets and every week subsequently while on the diets.

### Infections

*S. venezuelensis* and *N. brasiliensis* were maintained in NSG (NOD.Cg-Prkdcscid Il2rgtm1Wjl/SzJ) mice at the University of Chicago by subcutaneous injection of 10,000 larvae (*S. venezuelensis*) or 2,500 larvae (*N. brasiliensis*). The cycle was started anew every 3 mo (*S. venezuelensis*) or month (*N. brasiliensis*). *H. polygyrus* was maintained in C57BL/6J mice at the University of Chicago by oral gavage of 500 larvae. From 2 wk following infection, feces or cecal contents containing helminth eggs were spread on top of a Whatman paper, which was rolled up and placed into a beaker with 150 ml of water at 28°C with the feces-free end standing in the water. The hatching larvae were collected over 2–7 days, and the cycle was reinitiated. For experiments, mice were subcutaneously injected with 1,000 *S. venezuelensis* larvae and 2,000 *N. brasiliensis* larvae. The height of each of these pathogens is 8 days following injection, and B6 mice used in experiments cleared each infection 12–14 days following subcutaneous infection in our facility. Mice were administered an oral gavage of 200 *H. polygyrus* larvae; the acute phase of the infection is 14 days following administration, after which the infected mice are chronically infected.

Reovirus strain T1L was propagated as previously described (Bouziat et al., 2018). The virus was recovered using reverse genetics and plaque-purified. Virus stocks were prepared using cesium chloride gradient centrifugation and dialyzed exhaustively. Titers of virus stocks were determined by plaque assay. The genotype of T1L was confirmed by SDS–polyacrylamide gel electrophoresis. Mice were infected with 10^9^ PFU of T1L diluted in PBS by oral gavage and euthanized 48 h following infection.

*Y. pseudotuberculosis* (strain IP32777) was originally obtained from Igor Brodsky (University of Pennsylvania) and grown in 2XYT media supplemented with 2 μg/ml Irgasan overnight at 25°C with vigorous shaking. Mice were fasted overnight prior to oral gavage with 1 × 10^8^ CFU of *Y. pseudotuberculosis* and harvested 48 h following infection.

### Immunofluorescence staining

For whole-mount imaging, the corresponding small intestine segment was excised and cut longitudinally to expose the lumen. The intestines were immediately washed with ice-cold PBS, then pinned down lumen side-up, and fixed in 4% PFA for 2 h at room temperature (RT) followed by two consecutive quick washes with PBS to remove any remaining PFA. Intestines were then embedded in 4% low-melt agarose (GoldBio), and sectioned with a vibratome (125 µm, 0.8 mm/s, 0.8 mm) to obtain sections with single rows of intestinal villi. Floating tissue sections were washed on a platform shaker at RT 3 times for 15 min each in 0.5% Triton X-100 (Thermo Fisher Scientific) in PBS (PBS-T), then replaced with blocking buffer (5% BSA, 5% donkey serum, and 0.002% sodium azide in PBS-T), and placed back on the shaker for 3 h at RT. Once complete, samples were stained with the indicated primary antibody in blocking buffer, with shaking for 48 h at 4°C. After 3 consecutive 15-min washes in PBS-T, samples were stained with secondary antibody in blocking buffer, with shaking for 24 h at 4°C. Tissues were washed a final three times for 30 min each at RT to remove excess unbound secondary antibody and mounted in DAPI Fluoromount-G clear mounting media (Thermo Fisher Scientific). The following primary antibodies were used for immunofluorescence: goat polyclonal anti-LYVE-1 (1:200, AF2125; Thermo Fisher Scientific), rabbit polyclonal anti-LYVE-1 (1:200, Ab14917; Abcam), goat polyclonal anti-VE-cadherin (1:100, AF1002; R&D Systems), rabbit polyclonal anti-Prox1 (1:100, Ab101851; Abcam), rabbit monoclonal anti-Ki67 (1:50, Ab16667; Abcam). Alexa Fluor secondary antibodies donkey anti-goat 488, donkey anti-goat 568, donkey anti-rabbit 488, and donkey anti-goat 568 (1:500; Thermo Fisher Scientific) were used for visualization. Images shown were captured with a Leica SP5 Tandem Scanner Spectral 2-photon confocal microscope (Leica Microsystems, Inc.). Digital image files which the scoring was based off were taken with Olympus VS200 Research Slide Scanner (Olympus/Evident) with a Hamamatsu ORca-Fusion camera (Hamamatsu Photonics). Individual images were visualized using OlyVIA Viewer software (Olympus/Evident).

### Goblet cell and eosinophil scoring

For quantifying goblet cells and eosinophils, 5-μm paraffin cross sections of duodena from uninfected mice or mice infected with *S. venezuelensis* were subjected to periodic acid-schiff (PAS) or Hematoxylin and Eosin (H&E) staining, respectively, at the University of Chicago Histology Core and the slides were scanned using an Olympus slide scanner at 20 × magnification. To determine the percentage of goblet cells out of total epithelial cells, 10 villi per section (each representing a biological replicate) were analyzed for a total number of epithelial cells versus PAS-positive–only cells (goblet cells) using QuPath software: villus epithelium was outlined manually, and positive cell detection was achieved by setting the cell detection parameters to sigma = 1.5 µm, minimum area = 10 µm^2^, maximum area = 400 µm^2^, with intensity parameters having a threshold of 0.1 and maximum background intensity of 2. Goblet cells were determined by training the classifier on PAS-positive cells (Score compartment cell: DAB OD max, Threshold 1+ = 0.2, Threshold 2+ = 0.4, Threshold 3+ = 0.6). A minimum of 400 cells was counted per section, and the percentage of goblet cells was obtained by dividing the PAS-positive by the total epithelial cells. For quantifying eosinophils per villus, nucleated eosin–positive cells (eosinophils) over eosin-negative nonepithelial cells were counted manually. This was chosen over an automated method to account for intraepithelial immune cells also. Again, 10 villi per section, representing biological replicates, were scored. A minimum of 200 cells was counted per section, and the percentage of eosinophils was obtained by dividing nucleated eosin–positive cells (eosinophils) by eosin-negative nonepithelial cells.

### Fecal FFA measurements

8-wk-old mice were infected with ∼1,000 *S. venezuelensis* larvae or not. To determine whether infection impacted primary dietary fate absorption, a group of mice was switched to 60% calories from fat diet, while another remained on the chow diet 5 days after infection, and 8-day postinfection feces were collected and frozen at −80°C until analysis. This setup, rather than a short-term oil gavage regime, was chosen to circumvent any confounding effect stemming from potential differences in gastrointestinal transit time. FFAs were measured using a colorimetric FFA assay kit (MAK466; Sigma-Aldrich) according to the manufacturer’s protocol with the following adaptation: feces were weighed and dissolved in ([weight (mg)]×5) μl of 1× NP-40 Substitute Assay Reagent (10010303; Cayman Chemicals) and 1 × protease inhibitor cocktail-EDTA free (Roche). Nonsoluble material was briefly spun down, and samples were measured at 1:10 dilutions of the solubilized feces, with the standard curve being prepared in one part of 1× NP-40 Substitute Assay Reagent and nine parts assay buffer.

### Dexamethasone treatment

A dexamethasone emulsion was made by first making a sterile 100 mg/ml solution of dexamethasone (D4902; Sigma-Aldrich) in DMSO and then diluting it to a final 2.5 mg/ml in sterile corn oil. A vehicle control was final 2.5% DMSO in corn oil. 8-wk-old mice infected with ∼1,000 *S. venezuelensis* larvae or not were intraperitoneally injected with 100 μl of 2.5 mg/ml dexamethasone emulsion or vehicle control once daily on days 4–7 after infection. Intestines and feces were harvested on day 8 after infection.

### FISH

The FISH protocol was adapted from the literature (Salzman et al., 2002; Swidsinski et al., 2005). Briefly, intestinal tissue was harvested with removal of all mesentery attached to the bowel wall. Tissue was immediately fixed in 0.5 ml of methacarn (60% methanol, 30% chloroform, 10% CH_3_COOH) for 6 h at 25°C in an Eppendorf tube. Tissues were washed three times in 70% EtOH for 10 min each, then paraffin-embedded with the lumen facing the upright position. 5-µm-thick paraffin sections were deparaffinized with serial 10-min washes with 95% xylene and then 90% xylene with brief washes in distilled water between. The sections were rehydrated in successive 10-min 95% and 90% EtOH washes followed by a 10-min wash in distilled water. 40 μl of bacterial probe (EUB338: /5Alex594N/5′-GCTGCCTCCCGTAGGAGT-3′/3AlexF594N/; IDT) (Hu et al., 2021) diluted 1:150 in hybridization buffer (0.9 M NaCl, 20 mM Tris-HCl [pH 7.2], 0.1% SDS) warmed to 50°C was added directly over the sections, which were then covered with a plastic coverslip. Slides were incubated in a humidifying slide chamber for 3 h at 50°C. Following incubation, slides were washed two times for 10 min each in wash buffer (0.9 M NaCl, 20 mM Tris-HCl [pH 7.2]) with excess wash removed after the final wash with a Kimwipe. Slides were mounted in DAPI Fluoromount-G clear mounting media (Thermo Fisher Scientific) and covered with a glass coverslip for imaging.

### Histological analysis/scoring

Digital image .vsi files of whole-mount z-stacks were imported and analyzed with QuPath and ImageJ software. Tissue ID was blinded to the analyzer. Villus length was measured from the base of the crypt to the tip of the villus. Villus width was measured at the center of the villus. Lacteal length and surface area were measured from the base of the lacteal to its tip. Prox1+ or Ki67+ nuclei were costained with DAPI and were enumerated within the LYVE-1+ lacteal. Lacteal tight junction organization was categorized based on the following VE-cadherin staining patterns previously established by other groups (Baluk et al., 2007; Zhang et al., 2018; Bernier-Latmani et al., 2015): button-like junctions (discontinuous puncta), zipper-like junctions (continuous lines), and mixed/both (combination of discontinuous and continuous staining along the lacteal). For villus/lacteal length, villus width, and lacteal surface area, at least 100 nonconsecutive villi were scored per group. 10 nonconsecutive lacteals per mouse were used to enumerate Prox1+ or Ki67+ nuclei and analyze tight junction composition. Tight junction lengths were measured using the *Analyze particles* function in ImageJ as follows: individual VE-cadherin channel was converted to an 8-bit image, then adjusted to threshold. The region of interest (ROI) was defined with the freehand selection tool, and then, the particles within the ROI were analyzed with the size limits set to “1 to infinity” to minimize the contribution of background staining.

### Lipid uptake test

Following an overnight fast, 150 μl of olive oil (MilliporeSigma) was delivered via oral gavage for plasma and LN triglyceride measurements and Oil Red O staining. If indicated, 100 μl of 1% poloxamer 407 (Sigma-Aldrich) was administered via i.p. injection 15 min prior to olive oil gavage. 90 μl of blood was taken via cheek bleed into a tube containing 10 μl 0.5 mM EDTA at the following time intervals: 0, 30, 60, and 180 min after oil gavage (depending on the experiment). LNs were harvested after 3 h, as a pilot experiment showed that from 40 to 60 min after gavage, lipid concentrations reached an equilibrium in each small intestinal LN, and 3 h permitted us to report on the LNs and plasma of the same mice. LNs were weighed into preweighed 1.5-ml tubes on ice with 25 μl of the triglyceride assay kits’ diluted NP-40 substitute (Cayman Chemical Company) and further processed according to the manufacturer’s instructions and snap-frozen over dry ice and kept at −80°C until use. Plasma and LN triglyceride concentrations were measured using the Triglyceride assay kit according to instructions, and absorbance was measured at 540 nm. Fresh unfixed small intestine floating vibratome sections taken 3 h after olive oil gavage were stained with Oil Red O following the manufacturer’s protocol for the Oil Red O Staining Kit (Sigma-Aldrich). Floating duodenum vibratome sections were washed three times for 20 min each in PBS in Eppendorf tubes, then stained with BODIPY 493/503 C_3_ (1:500 in PBS) (D2191; Invitrogen) for 1 h with gentle shaking, and protected from light at RT. Tissues were then washed three times for 20 min each, stained with DAPI diluted into PBS for 10 min, washed with PBS for 15 min, then mounted.

### Recombinant IL-4 complex

The protocol that was used is described in detail and was adapted from Schneider et al. (2018). Briefly, 2 µg carrier-free recombinant IL-4 protein (404-ML-025/CF; R&D Systems) was incubated with 10 µg IL-4 antibody (clone: 11B11; Bio X Cell) per mouse for 30 min at RT. Volume was topped up with sterile PBS, then administered via i.p. injection once and then 48 h later, dissecting the mice 48 h after the last injection. PBS was administered to vehicle control mice.

### VEGF ELISAs

Each small intestine segment was removed, longitudinally opened, quickly rinsed with ice-cold PBS to remove luminal content, and then briefly dried with a Kimwipe, snap-frozen on dry ice, and kept at −80°C until processing. To extract protein from tissue, frozen tissue was homogenized with a tissue douncer in 300 μl cold radioimmunoprecipitation assay (RIPA) buffer (1% Triton X-100, 2% HEPES, 12% 2.5 M NaCl, 0.15% MgCl_2_, 0.04% 0.5 M EDTA, and 0.5% sodium deoxycholate) supplemented with protease inhibitor (1X) and PMSF (30 M). After homogenization, the homogenate was transferred to a clean Eppendorf tube and topped up with 300 μl of RIPA buffer, then incubated with gentle shaking at 4°C for 2 h. The homogenate was then spun down at 12,000 G for 20 min at 4°C and stored at −80°C. The tissue homogenate protein concentration was measured with a Pierce BCA protein assay kit (Thermo Fisher Scientific) per the manufacturer’s instructions. Samples were normalized to 50 µg/μl, then 2.5 mg of protein sample was loaded per well of each of the ELISA plates, and ELISAs were run per the manufacturer’s instructions: VEGF-A (DuoSet ELISA, R&D Systems), VEGF-B (Cusabio Biotech Co.), and VEGF-C (Cusabio Biotech Co.).

### Mucosal scrape (RNAseq)

The small intestine was removed from the mesentery, and Peyer’s patches were removed. The small intestine was divided into four quarters: the proximal ¼ was taken as the duodenum, ¼ of the middle of the small intestine was taken as the jejunum, and the terminal 2 inches were taken as the ileum. The tissues were cut longitudinally and gently washed with ice-cold PBS to remove any luminal contents. The tissue segments were immobilized, and the straight edge of a blade was used to scrape away the intestinal mucosa and submucosa from the muscularis. The muscularis was discarded, and the mucosa layers were snap-frozen on dry ice in 1.5-ml Eppendorf tubes with 0.5 ml of *RNAlater* (Sigma-Aldrich) and kept at −80°C until processing. Samples were defrosted on ice, and mucosal scrapes were transferred into 2-ml Bead Mill Tubes prefilled with ceramic beads and 1 ml cold TRIzol Reagent (Thermo Fisher Scientific). Samples were lysed with a bead beater, then transferred to a new tube, and processed according to the manufacturer’s instructions. Briefly, the samples were incubated at RT, then spun down at 4°C, and the supernatant was transferred to a new tube with chloroform to extract the RNA and spin down again. The RNA was precipitated by adding equal volumes of 70% EtOH and continuing the RNA purification process with the PicoPure RNA Isolation Kit (Thermo Fisher Scientific) according to the manufacturer’s instructions and eluting in 20 μl of the elution buffer. RNA concentration was measured with NanoDrop Microvolume Spectrophotometer (Thermo Fisher Scientific).

### Mucosal scrape RNAseq library prep

RNA samples were normalized to 200 ng/μl with DNAse/RNAse-free distilled water. 1 ng of RNA was used as the starting material for cDNA synthesis and tagmentation using the NEBNext Poly(A) kit (NEB) per the manufacturer’s instructions. The concentration of the eluted samples was calculated using a Qubit fluorometer (Thermo Fisher Scientific), and the average fragment length of 300–400 bp was confirmed with Bioanalyzer (Agilent). Samples were pooled at 10 nM and sequenced using paired-end 50-bp reads on a NovaSeq S2 flow cell at the University of Chicago Functional Genomics Core.

### BEC/LEC isolation from small intestine

Flt4^creERT2^TdTomato mice were administered dietary tamoxifen for 1 wk and harvested 2 wk after the tamoxifen regime had been terminated. Their small intestine was removed from the mesentery, and Peyer’s patches were removed. The small intestine was divided into four quarters: the proximal ¼ was taken as the duodenum, ¼ of the middle of the small intestine was taken as the jejunum, and the terminal 2 inches were taken as the ileum. The tissue was cut longitudinally and gently washed with ice-cold PBS to remove any luminal contents. The tissue was cut into 1-cm pieces and incubated in PBS with 1 mM DTT for 15 min at RT at 80 rpm to remove the mucus from the tissues. Tissues were then washed in PBS with 30 mM EDTA for 10 min at 37°C at 230 rpm with vigorous shaking after the incubation, then washed in fresh PBS, and incubated for 5 min at 37°C at 230 rpm. Tissues were washed again with PBS on a metal sieve and placed into a 6-well plate where they were finely chopped and digested in 2 ml RPMI containing 2% FCS, 1% HEPES, and 2 mg/ml collagenase VIII (Roche), 200 mg/ml DNase I (Roche) for 35 min at 37°C at 80 rpm. Tissues were physically disrupted by passing them 10 times through a 18G needle using a 3-ml syringe every 10 min, then 20 times at the completion of the digestion, as monitored for the appearance of single red cells under a benchtop fluorescence microscope. Digests were filtered and quenched in cold RPMI with 2% FCS.

### BEC-LEC sorting

250 BECs (live CD45-TER119-LIN-Podoplanin-CD31^+^) and 250 LECs (live CD45-TER119-Podoplanin+CD31^+^) were sorted from the duodenum, jejunum, ileum, and colon of uninfected and *S. venezuelensis*–infected Flt4cre^ERT2^ ROSA26^tdTomato^ mice. Live cells were sorted into 25 μl TCL buffer containing 1% 2-mercaptoethanol, immediately spun down, and snap-frozen on dry ice. Samples were kept at −80°C until further processing. All sorts were conducted at the University of Chicago Flow Cytometry Core using BD FACSAria Fusion Cell Sorter.

### BEC-LEC RNAseq library prep

Library preparation was performed as described previously (Esterházy et al., 2019). RNA was isolated using RNA Clean XP beads (Agencourt) on a magnetic stand. Reverse transcription primers were as follows: P1-RNA-TSO: Biot-rArArUrGr​ArUrArCrG​rGrCrGrAr​CrCrArCrCr​GrArUrNr​NrNrNrNrNrGrGrG, P1-T31: Biot-AATGATACGGCGACCACCGATCG31T, P1-PCR: Biot-GAATGATACGGCGACCACCGAT. RNA was eluted for 1 min in RT-cDNA synthesis mix 1 (0.5 μl P1-T31 [20 µM], 0.3 ml RNasin Plus [Promega], 1.5 μl 10 µM dNTP, 3.5 μl 10 µM Tris [pH 7.5]–0.5% IGEPAL CA-630 (Sigma-Aldrich), and 1.7 μl DNAse/RNAse-free distilled water) and pipetted up and down to mix. The eluted sample was then incubated for 3 min at 72°C, followed by 1 min on ice, and then, 7.5 μl of mix 2 was added (3 μl 5X RT buffer, 0.375 μl 100 mM DTT, 0.375 μl RNasin plus, 0.5 μl P1-RNA-TSO (40 µM), 0.75 μl Maxima RT Minus H (Thermo Fisher Scientific), 1.8 μl 5 M betaine (Sigma-Aldrich), 0.9 μl 50 mM MgCl_2_, and 0.175 μl DNAse/RNAse-free distilled water) and mixed well. Samples were placed in a thermocycler and subjected to the following PCR protocol: 42°C for 90 s, (50°C for 2 min, 42°C for 2 min) x10 cycles, 70°C for 15 min. The cDNA was then amplified using 13.5 μl cDNA, 20 μl KAPA HiFi 2x Mix, 1.5 μl P1-PCR, and 5 μl H_2_O. Samples were subjected to the following protocol: 98°C for 3 min, (98°C for 15 s, 67°C for 20 s, 72°C for 6 min) ×12 cycles, 72°C for 5 min. cDNA was cleaned up using RNA Clean XP beads and eluted in water. The concentration was calculated using a Qubit fluorometer, and the average fragment length of 1,500–1,800 was determined by Bioanalyzer. Samples were normalized to 0.1 ng/μl using DNAse/RNAse-free distilled water, and 2.5 μl cDNA was tagmented using Nextera XT Index Kit according to the manufacturer’s protocol, except that all volumes were used at 0.5x of the indicated volumes. The concentration of the eluted samples was calculated by Qubit. Samples were pooled at 10 nM and sequenced using paired-end 50-bp reads on a NovaSeq S2 flow cell at the University of Chicago Functional Genomics Core.

### RNAseq analysis

Raw fastq files were pseudoaligned to the mouse reference transcriptome M26 (GRCm39) using Kallisto (Bray et al., 2016). The gene counts were input to R, and transcripts per million was scaled by gene length. Initial filtering removed genes with expression under two cpm. Errant samples in the LEC and BEC sequencing experiment were identified by the lack of expression of their marker genes (*Prox1*, *Vegfr3*, and *Lyve1* for LECs, *Cd34* and *Kdr* for BECs) and were excluded from further analyses. Specific samples excluded are identified in Table S2. The un-normalized filtered gene counts were then used as input for the R package DESeq2 using the default parameters (Wald’s test) to compare genes differentially expressed across conditions or segments, correcting for replicates. The *apeglm* method was used for log fold change shrinkage to better visualize effect sizes. Genes with an adjusted P value (padj) ≤0.05 were considered significant for downstream studies. To generate segment- or condition-specific gene sets, the significant genes with log_2_ fold changes >1.5 or less than −1.5 and cutoff for both pairwise comparisons (i.e., Ileum LEC versus duodenum LEC, *Sv* 8 days after infection duodenum LEC versus noninfected duodenum LEC) were acquired using the R package DESEQ2. To perform and plot GSEA comparing two different segments or conditions, the R packages *fgsea* and *dplyr* were used with the following parameters: log_2_ fold change > 1.5 and less than −1.5, 10 genes per pathway minimum, 500 genes per pathway maximum, 10,000 permutations. GO Biological Process pathway (m5.go.bp.v2023.1.Mm.symbols.gmt) was used as a reference for the analysis. For figure space purposes, the GSEA plots shown were curated with removal of redundant or nonapplicable pathways. Pathways with the highest number of genes were kept over those with fewer genes. Heatmaps were generated using total DEG list (log_2_ fold changes >1.5 or less than −1.5) or from list of genes within collapsed GO Biological Process pathways using the R package pheatmap.

Venn diagrams comparing DEG list between datasets were generated using the R package Venn Diagram.

TF network and associated analysis were performed by putting all DEGs with a padj ≤ 0.05 into the ChEA3 search database (Keenan et al., 2019). Key TFs identified were confirmed to be expressed in sorted LECs as to having expression levels over 2 cpm.

Overrepresentation GO pathway analysis was performed by entering DEG list into several independent databases (https://bioinformatics.sdstate.edu/go/) (https://amigo.geneontology.org/amigo/landing) (Ge et al., 2020; Ashburner et al., 2000; Aleksander et al., 2023). For pie charts, pathways were collapsed into general like-categories. In several instances, genes were found to contribute to more than one pathway. To avoid biasing data, these genes were accounted for in each of the general pathways.

### Tissue RNA extraction and quantitative PCR

Whole intestinal tissue was harvested, and any attached mesentery was removed. The gut was then longitudinally cut to expose the lumen. The luminal contents were removed by washing the tissue in ice-cold PBS. Excess PBS was removed by briefly dabbing the tissue with Kimwipes, and then, the tissue was placed into 1.5-ml Eppendorf tubes with 0.5 ml of *RNAlater* and frozen over dry ice and kept at −80°C until processing. Samples were defrosted on ice, and tissue was transferred into 2-ml Bead Mill Tubes prefilled with ceramic beads and 1 ml cold TRIzol Reagent (Thermo Fisher Scientific). RNA was isolated using the TRIzol manufacturer’s protocol. cDNA was prepared with the SuperScript IV Reverse Transcriptase system and protocol (Invitrogen) using 1 μg RNA as input. cDNA was diluted with RNase-free water (1:10) upon completion. qPCR master mixes were prepared using Power SYBR Green (Invitrogen) containing 10 ng cDNA sample, 7.5 μl SYBR, 0.15 μl 10 µM forward and reverse primer mix, and water up to 14 μl. Samples were placed into 384-well plates in duplicate using a multichannel pipette and analyzed using an Applied Biosystems QuantStudio 6 Flex machine. Delta Ct was calculated by subtracting the average of duplicate values for each gene from the average duplicate values for the housekeeping gene *36b4* for each sample. Relative expression values were then calculated by the equation: 2^−∆CT^ × 10,000.

qPCR primer sequences are detailed in Table 1.

**Table 1.** qPCR primer sequence

Gene	Primer sequence	Sequence source
*36b4*	Fwd: 5′-GCC​GTG​ATG​CCC​AGG​GAA​GAC-3′	(Moest et al., 2013)
	Rev: 5′-CAT​CTG​CTT​GGA​GCC​CAC​GTT-3′	
*Gata3*	Fwd: 5′-CAA​CCT​CTA​CCC​CAC​TGT​G-3′	IDT
	Rev: 5′-GAT​GTC​CCT​GCT​CTC​CTT​G-3′	
*Gata1*	Fwd: 5′-CCC​AAT​GCA​CTA​ACT​GTC​AAA​C-3′	IDT
	Rev: 5′-ATC​TTT​CCT​CAT​GGT​CAG​TGG-3′	
*Mcp1*	Fwd: 5′-GAC​CCG​TAA​ATC​TGA​AGC​TAA​TGC-3′	IDT
	Rev: 5′-AAT​TAA​GGC​ATC​ACA​GTC​CGA​GTC-3′	
*F480*	Fwd: 5′-TTG​TAC​GTG​CAA​CTC​AGG​ACT-3′	PrimerBank
	Rev: 5′-GAT​CCC​AGA​GTG​TTG​ATG​CAA-3′	
*Muc2*	Fwd: 5′-ACA​AAA​ACC​CCA​GCA​ACA​AG-3′	(Pedicord et al., 2016)
	Rev: 5′-GAG​CAA​GGG​ACT​CTG​GTC​TG-3′	
*Il33*	Fwd: 5′-ACT​CCA​AGA​TTT​CGC​CG-3′	PrimerBank
	Rev: 5′-CAT​GCA​GTA​GAC​ATG​GCA​GAA-3′	
*Cd36*	Fwd: 5′-GAT​GAC​GTG​GCA​AAG​AAC​AG-3′	(Cifarelli et al., 2021)
	Rev: 5′-CAG​TGA​AGG​CTC​AAA​GAT​GG-3′	
*Ctgf*	Fwd: 5′-GTG​CCA​GAA​CGC​ACA​CTG-3′	(Hong et al., 2020)
	Rev: 5′-CCC​CGG​TTA​CAC​TCC​AAA-3′	
*Itgav*	Fwd: 5′-CTT​CAA​CCT​AGA​CGC​GGA​G-3′	(Li et al., 2023)
	Rev: 5′-CGG​TAA​AAC​TCC​ACG​GAG​AAG-3′	
*Cxcr2*	Fwd: 5′-TCC​TAA​CAC​TAG​ACC​CCA​AAC​ACT​C-3′	(Kawagoe et al., 2020)
	Rev: 5′-TTT​CTC​TCC​TCC​ACC​TCT​TCC​TT-3′	
*Emilin2*	Fwd: 5′-CCC​TGG​TGT​ATC​GGG​TAA​AC-3′	(Da Ros et al., 2022)
	Rev: 5′- ATG​TGG​TCT​TTG​GGA​CCT​TCT-3′	
*Il1b*	Fwd: 5′-GCC​CAT​CCT​CTG​TGA​CTC​AT-3′	(Willemze et al., 2019)
	Rev: 5′-AGG​CCA​CAG​GTA​TTT​TGT​CG-3′	
*Reg3a*	Fwd: 5′-ATT​GGG​CTC​CAT​GAT​CCA-3′	(Kurashima et al., 2021)
	Rev: 5′-AGA​TAA​TTC​AGC​ACA​TCG​GAG​TT-3′	
*Reg3b*	Fwd: 5′-ACT​CCC​TGA​AGA​ATA​TAC​CCT​CC-3′	(Kurashima et al., 2021)
	Rev: 5′-CGC​TAT​TGA​GCA​CAG​ATA​CGA​G-3′	
*Reg3g*	Fwd: 5′-TCC​ACC​TCT​GTT​GGG​TTC​AT-3′	(Pedicord et al., 2016)
	Rev: 5′-AAG​CTT​CCT​TCC​TGT​CCT​CC-3′	
*Defa20*	Fwd: 5′-CTT​GGC​CTC​CAA​AGG​AGA​TAG-3′	(Yue et al., 2021)
	Rev: 5′-AGA​CAC​TTG​TCC​TCC​TCT​CT-3′	
*Vegfc*	Fwd: 5′-CAG​GAC​AGG​GGA​CAG​TGT​AAA​ATT​TGC-3′	(Hong et al., 2020)
	Rev: 5′-TGG​CAT​GCA​TTG​AGT​CTT​TCT​CCA​C-3′	
*Prox1*	Fwd: 5′-TTC​TTT​TAC​ACC​CGC​TAC​CC-3′	IDT
	Rev: 5′-TTG​ACG​CGC​ATA​CTT​CTC​C-3′	

### Luminal content 16S sequencing

Small and large intestines were harvested and cut longitudinally. Luminal contents were gently removed, weighed in 1.5-ml Eppendorf tubes, and snap-frozen with liquid nitrogen. Samples were submitted to the University of Chicago, Duchossois Family Institute Microbiome Metagenomics Facility for library preparation, sequencing, and data analysis as previously described (Odenwald et al., 2023). Briefly, DNA was extracted using the QIAamp PowerFecal Pro DNA kit and quantified using Qubit. The V4–V5 region within 16S rRNA gene was amplified using universal bacterial primers—563F (5′-nnnnnnnn-NNNNNNNNNNNN-AYTGGGYDTAAA-GNG-3′) and 926R (5′-nnnnnnnn-NNNNNNNNNNNN-CCGTCAATTYHT-TTRAGT-3′), where “N” represents the barcodes, and “n” are additional nucleotides added to offset primer sequencing. The samples were sequenced on the Illumina MiSeq platform.

### Microbial DNA extraction from tissue and 16S qPCR to enumerate CFU from gene copy number

The protocol is described in detail and adapted from Barlow et al. (2020), Bogatyrev et al. (2020). Briefly, intestinal tissues were harvested and longitudinally opened. The luminal contents were gently removed, and then, the mucosa was scraped from the intestine and snap-frozen in 1 ml of DNA/RNA Shield solution (R1100-250; Zymo Research) over dry ice and kept at −80°C until use. Bacterial DNA was isolated from the mucosal scrapes using ZymoBIOMICS DNA Miniprep Kit (D4300; Zymo Research) per the manufacturer’s instructions. qPCR was set up in triplicate using 1.5 μl of template DNA, Power SYBR Green qPCR master mix (Invitrogen), 500 nM forward (UN00F2, 5′-CAGCMGCCGCGGTAA-3′) and 500 nM reverse (UN00R0, 5′-GGACTACHVGGGTWTCTAAT-3′) primers (Integrated DNA Technologies) with the reaction volume brought up to 15 μl with distilled water. Thermocycling program was as follows: initial denature (95°C for 15 min) and amplification (40 cycles, denature at 95°C for 15 s, annealing at 53°C for 10 s, and extension at 68°C for 45 s). To calculate the CFU per copy number, *Lactiplantibacillus plantarum* was serially diluted 1:10, and then, each dilution was split to be plated on MRS plates (69964-500G; Sigma-Aldrich) or have the DNA extracted as described above. Plate CFUs were paired with their respective cycle threshold values to generate a standard curve to extrapolate CFU from the copy number of the test samples.

### Broad-spectrum antibiotic treatment

The day before, day of, and day after *S. venezuelensis* infection, mice were gavaged with 200 μl of antibiotic solution containing 10 mg/ml vancomycin, 10 mg/ml metronidazole, 20 mg/ml ampicillin, and 20 mg/ml neomycin (“Abx,” each obtained from Sigma-Aldrich), dissolved in drinking water, and filter-sterilized. Gavage was used to achieve immediate and synchronized microbial depletion between mice. From 2 days after infection, low microbial load was maintained by placing mice on drinking water containing Abx (1 g/liter of ampicillin and neomycin, 0.5 g/liter vancomycin and metronidazole, 2 g/liter noncaloric sweetener Truvia to overcome avoidance of bitter taste), made available as sole and ad libitum liquid source in cages. Mice were harvested on day 9 postinfection. To confirm microbial depletion, fecal microbiome was extracted using QIAamp Fast DNA Stool Mini Kit (51604; Qiagen) and following the manufacturer’s protocol. Universal bacterial 16S primers used for qPCR were as follows: UNIF340: Fwd: 5′-ACT​CCT​ACG​GGA​GGC​AGC​AGT-3′ and UNIR514: REV: 5′-ATT​ACC​GCG​GCT​GCT​GGC-3′.

### Quantification and statistical analysis

For lacteal scoring, investigators were blinded to condition allocation during data analysis. RNAseq data were analyzed in R, including statistical analysis. All other data were analyzed with Prism software (GraphPad). Data are presented as average ± SD. Multivariate data between segments in a single condition were analyzed by applying one-way ANOVA with Šídák’s multiple comparisons test; comparison between two conditions was analyzed by two-tailed unpaired Student’s *t* test assuming a Gaussian distribution. Comparison of single variables between conditions was analyzed by applying two-way ANOVA with Turkey’s multiple comparisons test. P values or representative symbols are noted when differences are ≤0.1; all other differences were not found to be significant. *P < 0.05, **P < 0.01, ***P < 0.001, ****P < 0.0001.

### Online supplemental material

Fig. S1 provides detail and further quantification of lacteal morphology and tight junction composition analysis at homeostasis and when VEGF signaling is perturbed (supplemental to Fig. 1). Fig. S2 shows quantification of small intestinal lacteal morphology and tight junction composition after exposure to nonhelminth pathogens or after helminth clearance (supplemental to Fig. 2). Fig. S3 shows the sorting scheme and purity confirmation of LECs and BECs and further analysis of LEC bulk RNAseq (supplemental to Fig. 3). Fig. S4 shows further analysis of mucosal scrape bulk RNAseq and orthogonal validation of the type 2 response after infection with *S. venezuelensis* (supplemental to Fig. 4). Fig. S5 provides further detail on the microbiota changes after infection with *S. venezuelensis* and the impact of its combination with antibiotic treatment on epithelial gene expression (supplemental to Fig. 7). Table S1 summarizes the cpm and DEG of bulk RNAseq of LECs and BECs along the intestine of noninfected mice and mice infected with *S. venezuelensis*. Table S2 summarizes the cpm and DEG of bulk RNAseq of mucosal scrapes along the small intestine of noninfected mice and mice infected with *S. venezuelensis*.

## Supplementary Material

Table S1 shows bulk RNAseq of LECs and BECs along the intestine of NI mice and mice infected with *S. venezuelensis* 8 days earlier.

Table S2 shows bulk RNAseq of mucosal scrapes along the small intestine of NI mice and mice infected with *S. venezuelensis* 5 or 8 days earlier.

## Data Availability

RNAseq data are available at GEO under access codes GSE271711 (mucosal scrape) and GSE271712 (LEC and BEC RNAseq).
